# Co-designing the ‘LEAD’ strategy: A theory-informed stakeholder-led behavioural intervention to improve mass drug administration compliance for lymphatic filariasis elimination in an urban block of Odisha, India

**DOI:** 10.1371/journal.pntd.0014632

**Published:** 2026-09-16

**Authors:** Tanveer Rehman, Ananya Anurakta Pattanaik, Madhusmita Bal, Prakash Kumar Sahoo, Krushna Chandra Sahoo, Shubhashisha Mohanty, Sandipana Pati, Sanghamitra Pati

**Affiliations:** 1 ICMR-Regional Medical Research Centre, Bhubaneswar, Odisha, India; 2 Department of Health Research, Ministry of Health & Family Welfare, Health Technology Assessment India, Govt. of India, New Delhi, India; 3 National Vector Borne Disease Control Programme, Department of Health & Family Welfare, Bhubaneswar, Odisha, India; 4 State Institute of Health and Family Welfare, Government of Odisha, Bhubaneswar, Odisha, India; 5 ICMR Headquarters, New Delhi, India; Central University of Tamil Nadu, INDIA

## Abstract

**Introduction:**

Sustained compliance with Mass Drug Administration (MDA) is essential for interrupting transmission of Lymphatic filariasis (LF). However, behavioural and system-level barriers persist to hinder its uptake. We aimed to explore determinants of MDA participation using behavioural frameworks and to co-design a theory-informed, stakeholder-led intervention to enhance MDA compliance.

**Methods:**

A mixed-methods formative study was conducted between January and June 2025 in an LF-endemic block of Odisha, India. The quantitative component comprised a community-based KAP survey(n = 400) assessing knowledge, MDA coverage, and compliance. The qualitative component involved two focus group discussions and three key informant interviews with frontline health workers, community members, and programme supervisors to explore perceptions, barriers, and delivery challenges. Findings were mapped to the Theoretical Domains Framework (TDF) and the Capability–Opportunity–Motivation–Behaviour (COM-B) model to identify behavioural determinants and inform intervention design. Two stakeholder workshops were subsequently held to co-develop a contextually relevant implementation package.

**Results:**

Among 400 survey respondents (mean age 46 years; 52% female), MDA coverage was 81% (n = 322), while MDA compliance was 57% (n = 229). The main reasons for non-compliance included fear of side effects (40%), misconceptions about the disease, and mistrust of government medicines. Qualitative findings revealed barriers in **C**apability (limited awareness, inadequate communication skills), **O**pportunity (weak supervision, irregular supply, competing workloads), and **M**otivation (low risk perception, fear, misinformation). Stakeholders jointly developed the ‘LEAD’ strategy (**L**everaging existing community platforms, **E**ducation in schools, **A**wareness via digital media, and **D**elivery through Frontline Health Workers). LEAD integrates interpersonal communication by health workers, school-based education using children as health messengers, community mobilisation through self-help groups, and message reinforcement via digital media.

**Conclusion/Significance:**

The study identified multi-level behavioural and operational determinants influencing MDA uptake in an endemic Indian setting. The LEAD package will be evaluated in the next phase of our study for feasibility, acceptability, and effectiveness in strengthening India’s LF elimination efforts.

## Author summary

The main strategy to prevent transmission of Lymphatic filariasis (LF) is annual Mass Drug Administration (MDA), where all eligible individuals in endemic areas are offered antifilarial tablets free of cost. However, in many communities, people hesitate to take the medicine because of fear of side effects, low risk perception, and misinformation. Addressing these behavioural barriers is essential to meet its LF elimination targets. We conducted this study in a highly endemic district of Odisha, India, to systematically understand why people do not take the MDA drugs and how those barriers can be addressed through context-specific solutions. We used behavioural science frameworks to analyse data from community surveys, interviews, and discussions with health workers and programme managers. Through a series of participatory workshops with stakeholders, we then applied these insights to co-develop a locally relevant intervention package designed to improve MDA compliance. The process resulted in the creation of a multicomponent strategy that strengthens health workers’ communication skills, engages schools and self-help groups for community mobilisation, and uses digital media to reinforce key messages. This paper illustrates how a systematic, theory-informed, and stakeholder-driven approach can be used to design practical interventions for complex public health challenges, offering a transferable model for other neglected tropical disease programmes.

## Introduction

Lymphatic Filariasis (LF) remains a significant public health challenge globally and in India. As of 2024, India accounted for the largest share of the population requiring MDA in the South-East Asia region, with 306.9 million people across 161 implementation units still requiring treatment [1]. Millions remain at risk of chronic disability, social stigma and economic loss due to conditions such as lymphoedema and hydrocele [2]. The World Health Organization’s (WHO) Global Programme to Eliminate Lymphatic Filariasis (GPELF) has made substantial global progress through a two-pronged strategy: (i) interruption of transmission by achieving high coverage through annual Mass Drug Administration (MDA), and (ii) morbidity management and disability prevention (MMDP) for affected individuals [1]. India has aligned with these global strategies through the National Vector Borne Disease Control Programme (NVBDCP), which launched nationwide MDA in 2004 and integrated MMDP activities to provide care and support to patients [3]. Despite these efforts, India continues to face persistent challenges in achieving and sustaining high MDA coverage and compliance, the cornerstone strategy for interrupting LF transmission [4]. Odisha is among the severely affected states, with all 30 districts classified as LF-endemic. Since the initiation of the MDA programme, the state has demonstrated notable progress, reducing the average microfilaria rate from 6% in 2021 to 0.31% in 2024 [5]. Nonetheless, elimination targets remain unmet, as district-level eligibility to stop MDA is determined through Transmission Assessment Surveys (TAS) rather than aggregate microfilaria rates alone. Nationally, as of 2024, 141 districts across India have cleared TAS and no longer require MDA, while 43 districts are still awaiting TAS [1]. In Odisha, only nine districts had been declared eligible to stop MDA [5], underscoring that the gap between state-level microfilaria reduction and district-level elimination criteria reflects persistent implementation and survey bottlenecks rather than programme failure.

Non-compliance with MDA remains the single most critical barrier to elimination. Even when drug distribution coverage exceeds 80%, effective compliance (the proportion actually consuming the drug) often remains below 60% [2,4]. The reasons are complex and multifactorial: motivation (e.g., fear, stigma, low risk perception), opportunity (e.g., weak community engagement, lack of resources), and capability (e.g., inadequate training and low confidence among frontline health workers) [6]. At the community level, fear of side effects, low perceived disease risk, misconceptions about transmission, and mistrust of government-provided medicines remain widespread [2,5,7–10]. At the health system level, frontline health workers (FLHWs), including Accredited Social Health Activists (ASHAs), Anganwadi Workers (AWWs), and Auxiliary Nurse Midwives (ANMs), serve as the primary community interface for public health programmes in India. They are supported by Community Drug Distributors (CDDs), trained community volunteers who are recruited locally, receive a brief orientation prior to each MDA round, and are compensated per distribution cycle. Despite their pivotal role, both FLHWs and CDDs often lack adequate training, communication tools, and the confidence needed to effectively address community concerns, creating a persistent gap between drug distribution and actual drug consumption.

Traditional top-down information campaigns and one-time awareness drives have had limited impact, as they fail to address the deep-seated behavioural determinants of drug refusal and mistrust. Several context-specific strategies have shown potential to improve compliance when implemented through trusted community platforms. Community-based education delivered by trained FLHWs can enhance interpersonal communication, dispel misconceptions, and build trust [7,11]. In parallel, women’s self-help groups (SHGs) have been leveraged effectively to disseminate health information and promote health-seeking behaviour at the grassroots level, particularly in maternal health and nutrition interventions [7]. The participatory nature of SHGs and their strong social networks make them ideal vehicles for community mobilisation in LF elimination efforts. School-based education has also emerged as a powerful platform for health promotion in other public health programmes such as malaria control, tuberculosis prevention, and deworming initiatives. Engaging schoolchildren as “health messengers” has been shown to increase parental awareness and improve community-wide adoption of preventive behaviours [12,13]. More recently, the widespread use of smartphones and messaging applications has expanded the reach of health communication. Digital media tools such as short videos, WhatsApp messages, and community-based mobile campaigns have shown high acceptability and recall in both rural and peri-urban settings [14]. The integration of digital media with existing community structures therefore offers a scalable, low-cost approach to sustain awareness and motivation between MDA rounds.

Implementation science frameworks, such as the Theoretical Domains Framework (TDF) and the Capability–Opportunity–Motivation–Behaviour (COM-B) model, provide structured methods to diagnose behavioural determinants and design targeted interventions. These frameworks have been successfully applied in diverse health domains, including maternal health, vaccination, and chronic disease management, to identify context-specific barriers and develop feasible, evidence-based behaviour change strategies [15–18]. Yet, few studies have systematically applied behavioural theory to develop interventions addressing these determinants in LF elimination programmes. By applying the TDF and the COM-B model, we aimed to identify modifiable barriers to MDA uptake, prioritise targets for change, and collaboratively design a contextually tailored, theory-informed implementation strategy. The process described in this paper illustrates how participatory design can be operationalised to develop a practical, community-centred intervention to improve MDA compliance in a highly endemic area of Odisha, India.

We hypothesised that the persistent gap between MDA coverage and compliance in this setting is driven by modifiable behavioural and system-level factors that can be systematically identified using implementation science frameworks (TDF and COM-B) and addressed through a participatory, stakeholder-co-designed intervention. The objectives of this study were: (i) to identify and characterise behavioural determinants of MDA non-compliance; (ii) to map these determinants to the COM-B model; and (iii) to co-design a theory-informed, contextually adapted intervention package with local stakeholders.

## Methods

### Ethics statement

All study participants were adults. Permission was sought from all research participants through written consent. Ethical approval (ICMR-RMRC/IHEC-2024/024) for this study was obtained from the Institutional Human Ethics Committee (IHEC), and from the State Research and Ethics Committee. The research team exclusively managed all collected data, adhering to the ethical guidelines of the Indian Council of Medical Research (ICMR).

### Study design

This study represents the formative phase of a larger implementation research project aimed at strengthening community compliance with MDA for LF elimination. The present paper describes the systematic process undertaken to develop the intervention, beginning with evidence generation and culminating in the design of a theory-informed implementation package. We conducted a multi-phase mixed-methods study from January to June 2025. In the first phase, a community-based survey was carried out to assess the community knowledge of LF, coverage and compliance with MDA, and self-reported reasons for non-compliance. In the second phase, a qualitative study was conducted among multiple stakeholders to explore the perspectives of both implementers (e.g., FLHWs, CDDs, MDA supervisors) and beneficiaries (e.g., community members) regarding the delivery challenges, facilitators, and behavioural influences on MDA participation. In the third phase, findings from both phases were synthesised and systematically mapped onto the TDF and COM-B models to identify modifiable determinants of behaviour. These insights informed two stakeholder co-design workshops, during which participants applied the BCW approach to prioritise intervention functions, behaviour change techniques (BCTs), and delivery modes. The detailed steps followed in this systematic, theory-driven intervention development process are summarised in Fig 1.

**Fig 1:**
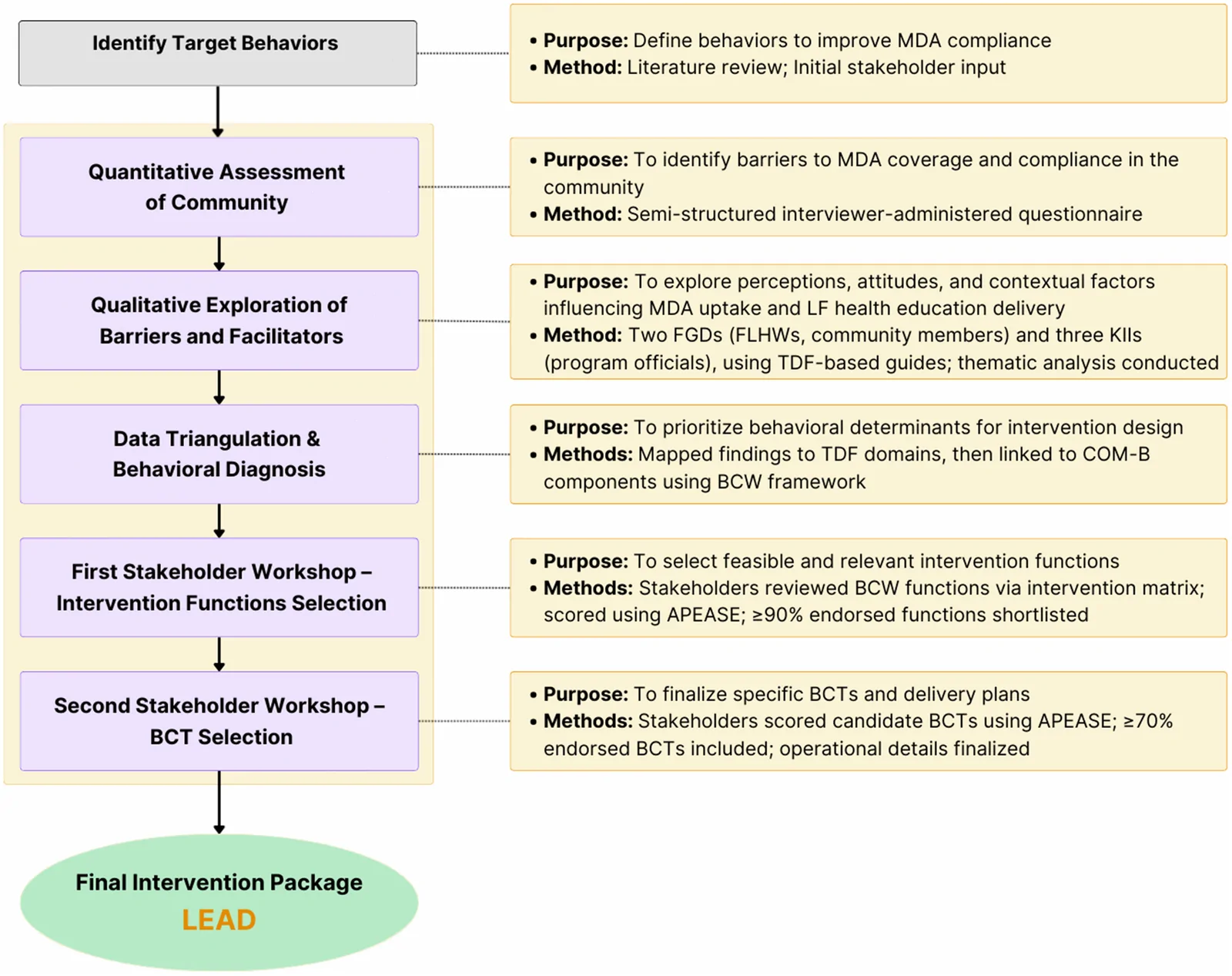
Summary of the systematic process to develop the intervention package. MDA – Mass Drug Administration; LF – Lymphatic Filariasis; FGD – Focus Group Discussion; FLHW – Frontline Health Worker; KII – Key Informant Interview; TDF – Theoretical Domains Framework; COM-B – Capability, Opportunity, Motivation–Behaviour; BCW – Behaviour Change Wheel; APEASE – Affordability, Practicability, Effectiveness/Cost-effectiveness, Acceptability, Side-effects/Safety, Equity; BCT – Behaviour Change Technique; LEAD – Leveraging existing community platforms through FLHWs, Education in schools, and Awareness via Digital media.

### Study settings

The study was conducted in the Athagarh Notified Area Council (NAC) of Cuttack district, Odisha, India. Despite ongoing programmatic efforts and government support, the area is characterised by persistently low compliance with MDA for LF. Multiple rounds of MDA have been implemented, yet the area continues to report suboptimal drug consumption and evidence of ongoing LF transmission [8]. Athagarh NAC is a highly endemic peri-urban and slum-dominated area where overcrowding, poor sanitation, and widespread mosquito breeding sites contribute to a disproportionate LF burden. The NAC is divided into 18 administrative wards, comprising 5,497 households with a total population of 27,304. As of 2024, 123 LF cases have been reported, with 18 new cases confirmed through night blood surveys conducted between 2021 and 2024, underscoring the urgency for intensified intervention.

Health services in the area include 13 Accredited Social Health Activists (ASHAs), 13 Anganwadi Workers (AWWs), two Auxiliary Nurse Midwives (ANMs), and one Sub-Divisional Hospital functioning as the primary referral facility. Outreach activities are supported through regular Urban Health Sanitation and Nutrition Days (UHSNDs). The area has 16 schools, but none formally deliver health education on LF or MDA. Additionally, 216 SHGs are operational, primarily focused on microfinance activities, with minimal involvement in health promotion. Before each annual MDA round, FLHWs (ASHAs, AWWs) and CDDs receive a two-day training by the State National Vector Borne Disease Control Programme (NVBDCP), focusing primarily on operational aspects such as household marking, record-keeping, coverage reporting, and drug distribution logistics. During the MDA campaign, ASHAs and AWWs are responsible for household-level drug distribution within their respective catchment areas, while CDDs assist with community mobilisation and door-to-door drug delivery. ANMs provide supervisory support and serve as the first point of contact for any adverse drug reactions, while district NVBDCP officials oversee coverage monitoring and report to state-level officers. However, these trainings provide limited guidance on interpersonal communication, community mobilisation, or addressing misconceptions. Public awareness campaigns typically occur one to two weeks prior to drug distribution, restricting opportunities for sustained behaviour change messaging.

### Study population, sampling, and data collection methods

#### Survey.

The community survey targeted adult residents aged 18 years and above who had lived in the study area for at least one year and expected to remain for at least two more years. Pregnant and lactating women and individuals with severe illness were excluded in accordance with national MDA guidelines, as they are ineligible to receive MDA drugs under NVBDCP recommendations. Given that a primary outcome was self-reported compliance (i.e., consumption of MDA drugs when last offered), their inclusion would have underestimated compliance, as non-consumption in these groups reflects programmatic ineligibility rather than behavioural refusal. A sample size of 400 participants was calculated based on an assumed MDA awareness level of 65% (±5%), with a 15% non-response allowance [19]. It is acknowledged that the systematic sampling across wards introduces a potential cluster design effect that was not accounted for in the sample size calculation; therefore, findings should be interpreted as descriptive and formative rather than inferential. A systematic random sampling approach was used across all 18 wards of Athagarh NAC. The total sample of 400 participants was allocated proportionally based on ward population (approximately 20–25 households per ward). Within each ward, every tenth household was selected, starting from a central landmark such as a school or temple. If a selected household was locked, the adjacent one was included. One eligible adult from each household was interviewed. A semi-structured questionnaire, adapted from the WHO-validated MDA coverage survey tools, was used to assess baseline knowledge, attitudes, practices, and barriers related to LF and MDA among the general population [20]. The instrument included sections on socio-demographic profile, knowledge and awareness of LF, preventive practices, MDA coverage and compliance, information channels, and communication preferences. The questionnaire was pre-validated and modified to suit the local context. The questionnaire was first drafted in English and subsequently translated into the regional language (Odia). Pre-testing was conducted with five adult community members purposively selected from a neighbouring ward not included in the main study, representing variation in age, sex, and educational background. This aimed to evaluate linguistic clarity and contextual relevance of the questionnaire. Feedback from the pilot was incorporated to revise ambiguous items and improve question flow. All data collectors received structured training by the study investigators on survey administration, ethical considerations, and community engagement.

#### Theoretical framework and model.

The TDF synthesises 33 behaviour change theories into 14 domains encompassing individual, social, and environmental influences such as knowledge, skills, beliefs, intentions, social norms, and contextual constraints [16]. The COM-B model conceptualises behaviour as the result of an interaction between three core elements: Capability (physical and psychological), Opportunity (social and environmental), and Motivation (reflective and automatic processes). Together, they provide a comprehensive basis for understanding determinants of behaviour and identifying suitable intervention functions. This combined framework, embedded within the Behaviour Change Wheel (BCW), has been successfully applied in diverse contexts, from school-based physical activity to dietary interventions for gestational diabetes prevention [17,18]. In this study, we applied TDF-guided quantitative and qualitative assessments to identify behavioural determinants of MDA uptake, mapped these findings to the COM-B constructs, and subsequently used the BCW approach to guide the co-design of a locally feasible, stakeholder-endorsed, multicomponent intervention package [21].

#### Qualitative interview.

A purposive maximum variation sampling strategy was used to capture diverse perspectives across three stakeholder groups: MDA implementers (ASHAs, AWWs, ANMs, CDDs), beneficiaries and community actors (community members, schoolteachers, ward councillors), and programme decision-makers and supervisors (district-level officers and MDA supervisors). Participants with experience of at least one MDA round were recruited with support from local healthcare providers, community volunteers, and the district health office. All participants were approached in person, and all consented to participate, with no refusals or dropouts. FGDs were conducted in a private community meeting space within Athagarh NAC, while KIIs took place at the offices of district NVBDCP officials and MDA supervisors. All interview settings were familiar, comfortable, and free from interruptions.

Two focus group discussions (FGDs) were conducted: one with FLHWs and CDDs, and another with community leaders, ward councillors, teachers, and community members (both those who accepted and those who declined MDA drugs). Discussions explored operational barriers, community perceptions, school-based dissemination, SHG involvement, and use of digital platforms for health communication. In addition, three key informant interviews (KIIs) were held with two district-level NVBDCP officers and a senior MDA supervisor to gain strategic insights into planning, training, monitoring, and systemic barriers. The FGD and KII guides were developed specifically for this study using open-ended questions with probes aligned to TDF domains (S1 file) [22]. These covered factors influencing MDA participation, including understanding of LF and MDA rationale; confidence and training to deliver health messages; perceived ability to influence community uptake; perceptions of one’s role; emotional drivers or barriers; infrastructure, logistics, and support systems; and the influence of family, community leaders, teachers, and peer networks. The face validity of the guide was reviewed independently by three authors with prior experience in TDF-based behavioural studies.

#### Stakeholder Engagement and Intervention Co-Design.

Two sequential multi-stakeholder consultation workshops were held in collaboration with the Odisha State NVBDCP and the Regional Health and Family Welfare Office. Participants were purposively selected from among qualitative study respondents who had provided detailed insights during interviews and discussions. Additional invitees included individuals repeatedly mentioned during the qualitative phase as influential community actors or potential health educators, such as schoolteachers, SHG leaders, and ward councillors. Of the 30 invited participants, 26 consented and actively participated. The workshops aimed to validate findings and collaboratively translate behavioural evidence into a locally relevant, theory-informed intervention strategy. Quantitative and qualitative data were triangulated and mapped to the TDF domains, which were then linked to the COM-B constructs (psychological/physical capability, social/physical opportunity, and reflective/automatic motivation). Using the BCW framework, nine candidate intervention functions (education, training, enablement, persuasion, modelling, environmental restructuring, incentivisation, coercion, restriction) were identified [15,23]. Each potential intervention function was systematically evaluated against the APEASE criteria (Affordability, Practicability, Effectiveness/Cost-effectiveness, Acceptability, Side-effects/Safety, and Equity). Functions achieving ≥90% consensus across all APEASE dimensions were shortlisted. Each function was rated using a five-point Likert scale (1 = strongly disagree to 5 = strongly agree) for each of the six APEASE criteria. Consensus was defined as ≥90% of participants rating ≥4 for all six criteria. Discrepancies were resolved through structured facilitated plenary discussion. In the second workshop, shortlisted functions were matched with appropriate Behaviour Change Techniques (BCTs) drawn from the BCT Taxonomy v1 [24]. Each BCT was illustrated with local examples and reassessed for contextual fit using the APEASE criteria. Endorsement scores were calculated as the proportion of participants (out of 26) rating a BCT as ≥4 on a five-point Likert scale. BCTs endorsed by ≥70% of participants (i.e., ≥ 18 out of 26 participants) were incorporated into the final intervention package.

Stakeholders then reviewed delivery methods, target audiences, and operational modalities to ensure feasibility within existing health system structures.

### Data management and analysis

Quantitative data were collected using the mobile-based Epicollect5 platform. Data were compiled, cleaned, and exported into Microsoft Excel for analysis. Descriptive statistics were used to summarise socio-demographic characteristics, LF-related knowledge, MDA compliance levels, and preferred sources of health information. Findings were deductively mapped to relevant domains of the TDF to inform behavioural diagnosis. All the FGDs and KIIs were conducted in Odia by two trained qualitative researchers (TR,AAP). Each session lasted approximately 60–90 minutes. No non-participants were present during any FGD or KII sessions. Field notes were recorded by the co-facilitator during and immediately after each session to capture contextual details and non-verbal cues. Data collection continued until thematic saturation was achieved, with no new themes emerging after the second FGD and third KII. Informed consent was obtained before audio recording. The audio recordings were transcribed verbatim in Odia and translated into English. To ensure accuracy, a second bilingual researcher independently reviewed 20% of translated transcripts against the original Odia transcripts; discrepancies were resolved by consensus. Transcripts were imported into NVivo 12 software for data management and coding procedures. Data were analysed using thematic framework analysis. A coding framework was developed based on the 14 domains of the TDF. Two trained researchers (TR,AAP) independently coded transcripts, achieving high inter-coder reliability (Cohen’s κ = 0.86; 95% CI: 0.83–0.89) [25]. Discrepancies were resolved through consensus, with arbitration by a third qualitative expert (KCS) when necessary. Each coded segment was categorised as a barrier or facilitator and tagged using participant identifiers (e.g., A1 for ASHA, T1 for teacher, S1 for MDA supervisor, O1 for program officer, N1 for ANM). A total of 182 meaningful utterances were coded, subsequently mapped to TDF domains and COM-B components, forming the basis for behavioural diagnosis.

In the first stakeholder workshop, the research team presented the synthesised behavioural diagnosis with TDF domains, mapped COM-B components, and candidate intervention functions from the BCW matrix. Stakeholders, including FLHWs, schoolteachers, NVBDCP officials, SHG leaders, and community volunteers, reviewed each function for contextual fit and health system feasibility. When multiple functions addressed a single COM-B component, participants independently scored each against the APEASE criteria. Scores were collated, and discrepancies were resolved through structured group discussion. Functions achieving ≥90% consensus across APEASE dimensions were shortlisted for further development. In the second stakeholder workshop, the shortlisted intervention functions were matched with potential BCTs from the BCW-linked taxonomy. Each BCT was described with a local example and assessed again using the APEASE criteria. Endorsement scores were calculated, and those meeting the ≥ 70% threshold were included in the final intervention package. To ensure accuracy and feasibility, the proposed delivery methods, target audiences, and operational details for each BCT were reviewed and confirmed by the stakeholders who contributed to the earlier discussions.

The reporting of qualitative methods and findings was guided by the Consolidated Criteria for Reporting Qualitative Research (COREQ) checklist. (S2 file)

## Results

### Participants characteristics

A total of 400 community members participated in the survey, with a mean (SD) age of 46.2 (13.8) years; 52% were female. Regarding education, 7% (n = 28) reported no formal education, while 35% (n = 141) had completed secondary education (9–10 years) (**Table 1**).

**Table 1:** Socio-Demographic characteristics of the survey participants (N = 400).

Variable	Category	Percentage
**Gender**	Male	48%
	Female	52%
**Age Group (years)**	18–40	39%
	41–60	44%
	>60	17%
**Occupation**	Regular Paid Job (Govt.)	4%
	Regular Paid Job (Private)	11%
	Irregular Paid Job	10%
	Not Working	13%
	Homemaker	38%
	Others	24%
**Education Level Completed**	No Formal Education	7%
	Primary (1–5 standard)	18%
	Middle (6–8 standard)	16%
	Secondary (9–10 standard)	35%
	Higher (> 10^th^ standard)	24%

A total of 20 individuals participated in the qualitative component, including two FGDs and three KIIs. FGD 1 included nine FLHWs (ASHAs, AWWs, ANM, and CDDs) with a median (IQR) age of 37 (25–44) years, 10 years of education, and 14 years of work experience. FGD 2 included eight community members (ward councillors, school teachers, and MDA recipients/non-recipients), of whom 50% were women, with a median age of 45 (26–58) years and 11 (8–16) years of education. Three KIIs were conducted with one MDA supervisor and two district NVBDCP officials (Table 2).

**Table 2:** Socio-Demographic Characteristics of Focus Group and KII Participants (N = 20).

Participant Group	Role Types	Sample Size (N)	Women n (%)	Median age (in years) (IQR)	Median years of education (IQR)	Median work experience (years, IQR)
FGD 1: FLHWs/CDDs	3 ASHAs 3 AWWs 1 ANM 2 CDDs	9	7 (78%)	37 (25-44)	10 (9-12)	14 (10-17)
FGD 2: Community Members	2 Ward councillors, 3 MDA Recipients/ non-recipients, 3 school teachers	8	4 (50%)	45 (26-58)	11 (8-16)	–
KII	1 MDA Supervisor, 2 District NVBDCP Officials	3	1 (33%)	43 (41-45)	16 (16-17)	17 (14-17)

*ASHA – Accredited Social Health Activist; AWW – Anganwadi Worker; ANM – Auxiliary Nurse Midwife; CDD – Community Drug Distributor; KII – Key Informant Interview; FGD – Focus Group Discussion; NVBDCP – National Vector Borne Disease Control Programme; IQR – Inter-quartile Range*

Twenty-six stakeholders participated in the co-design workshops, representing FLHWs (n = 10), schoolteachers (n = 4), SHG leaders and community volunteers (n = 6), NVBDCP officials (n = 2), and public health researchers (n = 4) with expertise in implementation science, health systems, filariasis, socio-behavioural health, and communication. Of these, 65% (n = 17) were female. The mean age was 41.2 years (SD ± 7.8), with a median of 12.5 years of professional experience (IQR: 10–16) and 14 years of formal education (IQR: 12–16) (**Table 3**).

**Table 3:** Characteristics of Stakeholder Workshop Participants (N = 26).

Variable	Category	Percentage
**Gender**	Male	34.6%
	Female	65.4%
**Occupation**	ASHA/ANM	38.5%
	School Teachers	15.4%
	SHG Leaders/Local Volunteers	23.1%
	NVBDCP Officials	7.7%
	Researchers (Implementation/Public Health)	15.4%

*ASHA – Accredited Social Health Activist; ANM – Auxiliary Nurse Midwife; SHG – Self-Help Group; NVBDCP – National Vector Borne Disease Control Programme*

### Quantitative survey findings- Coverage, Knowledge Gaps, and Beliefs About LF

Among respondents, MDA coverage was 81% (n = 322), while MDA compliance was 57% (n = 229). Knowledge gaps were evident, with 33% (n = 130) unaware of the cause of LF and 22% (n = 85) believing it to be hereditary. Among non-compliant individuals (n = 236), key reasons included fear of side effects (40%), perceived lack of necessity (36%), missed drug distribution (13%), and pre-existing health conditions (8%). Compliance varied by education level, highest among those with no formal education (75%) and lowest among those with higher education (44%). These findings were mapped to TDF domains, highlighting gaps in knowledge and beliefs about consequences.

### Qualitative insights and triangulation- barriers, enablers, and prioritized COM-B domains

Thematic analysis identified multi-level barriers and enablers to MDA implementation. At the community level, misconceptions about LF transmission and fear of side effects were major barriers to compliance. At the implementer level, ASHAs and teachers reported competing workloads, limited training, and unclear roles, which reduced confidence and motivation. At the health system level, delayed IEC material distribution, weak coordination, and poor integration of MDA communication into existing platforms were key challenges. Suggested improvements included earlier planning, strengthened training, and better use of community platforms to enhance awareness.

Triangulation of qualitative and quantitative findings yielded ten prioritised TDF domains and deductively mapped to COM-B components. **Table 4** presents integrated results with illustrative quotes and their mapping to COM-B components. These mapped domains provided the foundation for the behavioural diagnosis, guiding the design of intervention functions within the BCW.

**Table 4:** Mapping of determinants from qualitative & quantitative survey findings, to the Theoretical Domains Framework (TDF) and COM-B model.

Determinants	Domain (Definition)*	COM-B System
Lack of community knowledge about LF (Barrier)	**1. Knowledge** (An awareness of the existence of something)	**Psychological Capability**
Need for early education (Facilitator)		
Parental knowledge gap (Barrier)		
Difficulty handling skepticism under time pressure (Barrier)	**2. Skills** (An ability or proficiency acquired through practice)	**Psychological Capability**
Using personal testimony (Facilitator)		
Confidence in community influence (Facilitator)	**3. Beliefs About Capabilities** (Acceptance of the truth, reality, or validity about an ability, talent, or facility that a person can put to constructive use)	**Reflective Motivation**
Leveraging peer influence (Facilitator)		
Creative pedagogy confidence (Facilitator)		
Fear of side effects (Barrier)	**4. Beliefs About Consequences** (Acceptance of the truth, reality, or validity about outcomes of a behaviour in a given situation)	**Reflective Motivation**
Refusal due to side effect fears (Barrier)		
Limited teacher role awareness (Barrier)	**5**.**Social/Professional Role & Identity** (A coherent set of behaviours and displayed personal qualities of an individual in a social or work setting)	**Reflective Motivation**
Trusted community messengers (Facilitator)	**6. Social Influences** (Those interpersonal processes that can cause individuals to change their thoughts, feelings, or behaviours)	**Social Opportunity**
Influence of local trusted figures (Facilitator)		
Youth engagement (Facilitator)		
Student-to-parent influence (Facilitator)		
Physical access challenges (Barrier)	**7. Environmental Context & Resources** (Any circumstance of a person's situation or environment that discourages or encourages the development of skills and abilities, independence, social competence, and adaptive behaviour)	**Physical Opportunity**
Lack of visibility compared to other programs (Barrier)		
Existing platforms for outreach (Facilitator)		
Need a record system to observe LF HE sessions (Barrier)	**8. Behavioural Regulation** (Anything aimed at managing or changing objectively observed or measured actions)	**Psychological Capability/ Physical Opportunity**
Multi-channel reminders (Facilitator)		
Parent-teacher group utility (Facilitator)		
Community resistance (Barrier)	**9. Emotion** (A complex reaction pattern, involving experiential, behavioural, and physiological elements, by which the individual attempts to deal with a personally significant matter or event)	**Automatic Motivation**
Professional pride (Facilitator)		
Strategy ownership (Facilitator)	**10. Intentions** *Conscious decision to perform behaviour*	**Reflective Motivation**
Political engagement (Facilitator)		

LF – Lymphatic Filariasis; MDA – Mass Drug Administration; COM-B – Capability, Opportunity, Motivation–Behaviour; TDF – Theoretical Domains Framework

### Stakeholder perspectives on LF prevention- barriers, enablers, and recommendations

In the first workshop, stakeholders reviewed nine candidate BCW functions. Education, Training, Enablement, Persuasion, and Modelling were endorsed as the most feasible and impactful. Environmental Restructuring was considered valid but limited by resources, while Incentivisation, Coercion, and Restriction were deprioritised due to low feasibility and acceptability. The prioritized linkages between COM-B sources of behaviour, TDF domains, and selected intervention functions are summarized in Fig 2.

**Fig 2:**
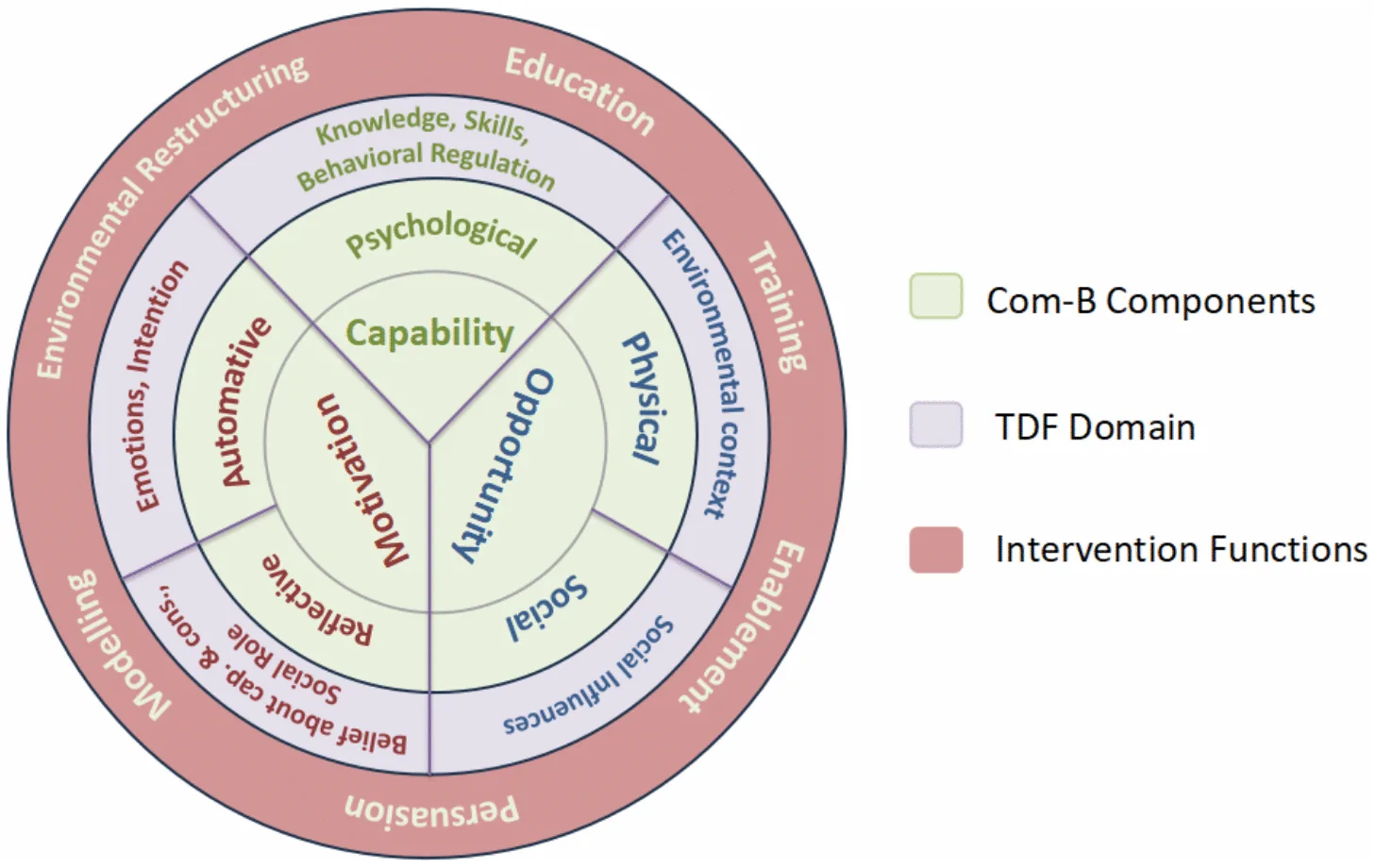
Prioritized COM-B, TDF domains, and intervention function mapping using the Behaviour Change Wheel (BCW). Cap. – capability; Cons. - consequences.

The second workshop finalised 19 BCTs judged practical, culturally appropriate, and aligned with local platforms. Strongly endorsed techniques included BCT 5.1 (*Information about health consequences*), BCT 4.1 (*Instruction on how to perform the behaviour*), BCT 6.1 (*Demonstration of the behaviour*), BCT 2.3 (*Self-monitoring of behaviour*), and BCT 7.1 (*Prompts/cues*). Stakeholders also co-designed the operationalisation of these techniques, specifying delivery methods, target audiences, and frequency (**Table 5**).

**Table 5:** Matrix of links between TDF domains, COM-B model intervention functions, and behaviour change techniques.

**TDF & COM-B**	**Intervention Functions**	**Behaviour Change Techniques**	**Description of Intervention Strategy**
Knowledge- *Psychological Capability*	Education, Enablement	4.1. Instruction on how to perform behaviour 5.1. Information about health consequences 5.3. Information about social/ environmental consequences	• Guide FLHWs on how to deliver simple infographics and jargon-free MDA messages for the community members. • Educate the community about LF transmission, MDA benefits, and consequences of non-compliance. • Highlight the community-level impact of MDA non-compliance.
Skills- *Psychological Capability*	Training, Enablement	4.1. Instruction on how to perform behaviour 6.1. Demonstration of behaviour 8.1. Behavioural practice/rehearsal	• Provide structured training and peer-led role-play exercises during monthly review meetings to improve health education delivery. • Provide clear steps on conducting health education using flipbooks, brochures, etc. • Practice sessions for FLHWs on responding to difficult questions about side effects, etc.
Beliefs About Capabilities- *Reflective Motivation*	Persuasion, Enablement	13.1. Identification of self as role model 15.1. Verbal persuasion about capability 15.3. Focus on past success	• Orient nodal teachers through workshops and provide tools (videos, posters) that highlight their role in LF awareness promotion. • Encourage FLHWs by emphasizing past success in increasing compliance and trust. • Position trusted FLHWs and teachers as role models in the community.
Beliefs About Consequences- *Reflective Motivation*	Persuasion, Modelling	5.1. Information about health consequences 9.1. Credible source 9.2. Pros and cons 13.1. Identification of self as role model	• Emphasize LF prevention via annual MDA; counter misconceptions about harm. • Use trusted figures (e.g., FLHWs, teachers, doctors) to reassure about MDA safety. • Clarify benefits vs. rare side effects in community sessions and brochures. • FLHWs demonstrating MDA drug consumption to show trust and safety.
Social/Professional Role & Identity- *Reflective Motivation*	Education, Modelling	4.1. Instruction on how to perform behaviour 5.3. Information about social/ environmental consequences 13.1. Identification of self as role model	• Clear training on their expected role in LF education during routine activities (e.g., weekly once in UHSND, Kishori Baithak, schools, SHG meetings) • Highlight how their engagement helps protect community health. • Encourage teachers and FLHWs to embrace their role as MDA educators and influencers.
Social Influences- *Social Opportunity*	Modelling, Enablement	3.1. Social support (unspecified) 6.1. Demonstration of behaviour 15.3. Focus on past success	• Leverage SHGs, ASHAs, and Anganwadi workers for message dissemination and peer support. • Trusted health workers and teachers to demonstrate drug consumption or advocacy techniques. • Highlight community adoption (e.g., “most of your ward has already taken MDA”).
Environmental Context & Resources- *Physical Opportunity*	Environmental Restructuring	7.1. Prompts/cues 12.1. Restructuring the physical environment 12.2. Restructuring the social environment	• Posters, wall markings, and digital reminders during MDA season to increase visibility. • Use existing platforms (UHSNDs, SHG meetings, schools) for regular LF awareness sessions. • Organize drug administration to reach people at workplaces or during peak times.
Behavioural Regulation- *Psychological Capability/ Physical Opportunity*	Enablement	1.4. Action planning 2.3. Self-monitoring of behaviour 7.1. Prompts/cues 12.2. Restructuring the social environment	• Plan LF health education during routine events (e.g., UHSNDs, ANC check-ups). • Use simple logbooks or mobile checklists to track sessions and follow-ups. • Posters, digital messages, and audio content serve as reminders for both community and FLHWs. • Use parent-teacher WhatsApp groups for scheduled message sharing weekly.
Emotion- *Automatic Motivation*	Persuasion	5.6. Information about emotional consequences 6.1. Demonstration of behaviour 11.2. Reduce negative emotions	• Clarify that refusal due to fear may cause long-term regret if LF develops. • Show respected figures (e.g., Councillors, Chairman) taking MDA tablets in front of the community to alleviate fear. • Use testimonials or videos showing community members successfully taking MDA without adverse effects.
Intentions- *Reflective Motivation*	Education, Persuasion	1.4. Action planning 5.1. Information about health consequences 5.3. Information about social/ environmental consequences 10.4. Social reward 13.1. Identification of self as role model	• Explain how collective community involvement (SHGs, schools, ward members) can improve MDA uptake. • Highlight how community-wide support fosters safer neighbourhoods and healthier families. • Collaboratively plan specific LF health education activities with SHGs, teachers, and ward members, assigning clear roles and timelines. • Recognize and reward active participation by FLHWs, SHGs, and schools in health education. • Present MDA-compliant patients as real-life role models (“champions”) to inspire community trust and intent.

*COM-B – Capability, Opportunity, Motivation–Behaviour; TDF – Theoretical Domains Framework; FLHW – Frontline Health Worker; MDA – Mass Drug Administration; LF – Lymphatic Filariasis; ANC – Antenatal Care; UHSND – Urban Health Sanitation and Nutrition Day; SHG – Self-Help Group.*

### Co-design of the LEAD intervention package from perceptions to practice

The culmination of this process was the development of the LEAD strategy. We used the term “LEAD” as it symbolises its central aim of “leading” communities towards improved MDA compliance by strengthening interpersonal communication, fostering participatory engagement, and reinforcing awareness through multi-platform behavioural approaches.

**L** – Leveraging existing community platforms: Utilises SHGs, schools, and local governance structures for sustained awareness and mobilisation.

**E** – Education in schools: Empowers schoolchildren as health messengers through interactive learning modules and parent–child communication activities.

**A** – Awareness via Digital media: Employs short, locally produced videos and WhatsApp messages for repeated, scalable dissemination of key messages.

**D** – Delivery through Frontline Health Workers: Strengthens FLHW capacity through tailored communication training and supportive supervision.

### Description of the LEAD intervention package

A culturally adapted printed brochure was developed as a visual and interactive job aid for FLHWs and teachers, using simple Odia language and illustrations to support comprehension across literacy levels and facilitate interpersonal communication. The brochure includes pictorial depictions of LF transmission, drug intake steps, and myth-busting messages (e.g., “Healthy people must also take the tablet”). The reverse side incorporates a session-tracking tool to record session details, promoting self-monitoring and accountability (S3 File). To reinforce in-person communication, digital materials such as short videos, infographics, and WhatsApp messages were designed using local voices to enhance credibility and relatability. This integrated approach aimed to ensure consistent messaging and sustained engagement across community and school settings while minimising additional workload for implementers (Table 6). This multi-platform strategy is carefully designed to maximise message reach and frequency, leveraging existing community and school engagement platforms while minimising additional workload for FLHWs and teachers (Table 6).

**Table 6:** Target behaviours and behavioural specification for improving MDA compliance.

Behavioural targets	Behavioural Specifications			
	WHO	WHAT	WHERE	WHEN
**Community-based education through FLHWs**	FLHWs (ASHAs, AWWs, ANMs, SHGs)	Deliver simple, repeated LF health messages using brochures, flipbooks, and interpersonal communication; address myths/fears; encourage drug intake during MDA	Community platforms: UHSND sessions, SHG meetings, Kishori Baithaks, ward meetings, home visits	Twice a month for 6 months, integrated with routine outreach sessions and intensified before/during MDA round
**School-based education – Children as health messengers**	Nodal school teachers	Conduct awareness sessions during morning assembly; organise quizzes/poster competitions; encourage students to carry messages home to parents	Schools (morning assembly, classroom sessions, parent–teacher meetings)	Twice a month for 6 months, with added reinforcement just before MDA round
**Digital messaging for reinforcement**	FLHWs, school teachers, SHG/youth leaders, ward councillors	Share short videos, posters, and WhatsApp messages on LF, importance of MDA, and reassurance on side effects	WhatsApp community/parent groups, local digital platforms	Twice a month for 6 months, aligned with community/school sessions and MDA calendar

*FLHW – Frontline Health Worker; MDA – Mass Drug Administration; LF – Lymphatic Filariasis; ASHA – Accredited Social Health Activist; AWW – Anganwadi Worker; ANM – Auxiliary Nurse Midwife; UHSND – Urban Health Sanitation and Nutrition Day; SHG – Self-Help Group*

## Discussion

The present formative implementation study systematically identified and addressed behavioural barriers to MDA for LF in a high-burden setting of Odisha, India, using the TDF, COM-B model, and structured stakeholder engagement. Despite high drug distribution, consumption remained suboptimal, driven by low disease awareness, fear of side effects, perceived lack of necessity, and limited FLHW communication capacity. These barriers are consistent with evidence from other endemic settings [26–28] and have been documented across multiple Indian states including Odisha [4,29], Uttar Pradesh [30], and Tamil Nadu [31], underscoring that they reflect a broader national challenge requiring theory-informed behavioural strategies rather than drug distribution alone. Fear of adverse effects, rooted in community misinformation, was a particularly dominant determinant, as similarly reported by Krentel et al. (2016) [28] across other NTD programmes. The intervention therefore prioritised trusted local messengers including FLHWs, SHGs, teachers, and youth clubs to deliver repeated, tailored health education, supported by evidence that culturally trusted actors improve intervention acceptance [32,33]. Operational constraints among FLHWs, including competing priorities and limited health education capacity, were addressed by integrating LEAD into existing platforms such as UHSNDs, Kishori Baithaks, and maternal–child health events, minimising additional workload and avoiding parallel delivery structures. This integration supports the long-term sustainability and scalability of LEAD within existing health system infrastructure, without requiring substantial additional resources. The cost implications of repeated behaviour change campaigns and monitoring activities will be formally assessed in the subsequent evaluation phase. Compared with conventional MDA strategies such as mass media campaigns or door-to-door delivery, LEAD offers additional advantages by systematically addressing behavioural determinants, co-designing interventions with stakeholders, and leveraging trusted local messengers for personalised and repeated engagement.

Supplementary tools, including job aids, reminder mechanisms, and simplified message templates, were designed to strengthen behavioural regulation among FLHWs and improve consistency of message delivery [21]. The stakeholder engagement process proved central to ensuring the contextual and cultural relevance of the intervention. Active participation of health officials, FLHWs, teachers, and community representatives validated intervention content and optimised delivery frequency, messaging tone, and integration points. This participatory co-design approach is supported by implementation science literature as a key enabler of adoption, acceptability, and sustainability [34–36]. Our study demonstrates how theory-driven frameworks can effectively translate into pragmatic, context-specific intervention tools when informed by local expertise and community voices. The multicomponent strategy addresses key behavioural domains identified in the TDF and links them with delivery mechanisms grounded in local health system capacities. These findings are consistent with guidance emphasising the role of behavioural theory in implementation practice [15,37]. Studies have demonstrated that interventions emphasising community-based education and local co-design lead to improved health knowledge and program uptake. Yet, few studies have comprehensively applied behaviour change frameworks such as the TDF and the COM-B model in designing such interventions. Where employed, these frameworks have provided valuable insights into context-specific determinants and have guided the development of more tailored, effective strategies [38,39].

Several limitations should be acknowledged. The findings are context-specific, derived from a single peri-urban area (Athagarh NAC) within one endemic district of Odisha; generalisability to rural, tribal, or other state settings should be interpreted with caution. The survey sample size was calculated for simple random sampling without incorporating a cluster design effect, which may marginally underestimate standard errors; findings should therefore be interpreted as descriptive and formative rather than inferential. The qualitative component comprised one FGD per stakeholder group, which was appropriate given the small, geographically defined study area and limited pool of eligible participants; however, this may not achieve full thematic saturation in larger or more heterogeneous settings. Stakeholder recruitment through existing professional networks may have introduced selection bias. Finally, as a formative study, findings are based on self-reported perceptions rather than observed behavioural outcomes. In the next phase, the LEAD package will be tested to evaluate its feasibility, acceptability, and effectiveness in improving MDA coverage, compliance, and community trust.

## Conclusion

The study identified key behavioural and system-level barriers to MDA compliance, including persistent knowledge gaps, fear of side effects, and limited communication capacity among health workers. Guided by TDF and COM-B, these determinants were systematically analysed and translated into a stakeholder co-designed, multicomponent intervention, integrating community-based education, school engagement, and digital reinforcement. It is important to note that the present study represents the intervention development phase; no claim is made regarding effectiveness, which will be rigorously assessed in the subsequent evaluation phase. Future evaluation will determine its feasibility, acceptability, and effectiveness in enhancing MDA uptake in our study.

## Supporting information

S1 File Draft of TDF-based qualitative interview questionnaire. (docx) (DOCX)

S2 File COREQ Checklist. (DOCX)

S3 File Filariasis Poster. (pdf) (PDF)

S4 File Summary of themes mapped to TDF domains and COM-B components (Extended version of Table 4). (DOCX)

## Abbreviations

LF: Lymphatic Filariasis
WHO: World Health Organization
GPELF: Global Programme to Eliminate Lymphatic Filariasis
MDA: Mass Drug Administration
MMDP: Morbidity Management and Disability Prevention
NVBDCP: National Vector Borne Disease Control Programme
FLHW: Frontline Health Worker
CDD: Community Drug Distributor
SHG: Self-Help Group
LEAD: Leveraging existing community platforms through FLHWs, Education in schools, and Awareness via Digital media
TDF: Theoretical Domains Framework
COM-B: Capability, Opportunity, Motivation–Behaviour
BCW: Behaviour Change Wheel
NAC: Notified Area Council
PPS: Probability Proportional to Size
ASHA: Accredited Social Health Activist
AWW: Anganwadi Worker
ANM: Auxiliary Nurse Midwife
UHSND: Urban Health, Sanitation and Nutrition Day
FGD: Focus Group Discussion
KII: Key Informant Interview
CI: Confidence Interval
IQR: Interquartile Range
APEASE: Acceptability, Practicability, Effectiveness, Affordability, Side-effects, Equity
NTD: Neglected Tropical Disease

## References

[1] WHO. Lymphatic filariasis: fact sheet. https://www.who.int/news-room/fact-sheets/detail/lymphatic-filariasis 2023.

[2] Ratna P, Sinha A, Pati S, Sahoo PK. Factors influencing implementation of mass drug administration for lymphatic filariasis elimination: a mixed-method study in Odisha, India. Front Pharmacol. 2024;15:1297954. doi: 10.3389/fphar.2024.1297954 38414733 PMC10897020

[3] PIB I. Campaign to target 63 endemic districts across Bihar, Jharkhand, Karnataka, Odisha, Telangana, and Uttar Pradesh. https://www.pib.gov.in/PressReleasePage.aspx?PRID=2044057 2024.

[4] Babu BV, Mishra S. Mass drug administration under the programme to eliminate lymphatic filariasis in Orissa, India: a mixed-methods study to identify factors associated with compliance and non-compliance. Trans R Soc Trop Med Hyg. 2008;102(12):1207–13. doi: 10.1016/j.trstmh.2008.05.023 18632125

[5] NHM O. Replicable practices to improve quality of mass drug administration against lymphatic filariasis. 2022. https://nhm.gov.in/New-Update-2024-26/Innovation-Summit/Odisha_Improving%20Mass%20Drug%20Administration_9th%20National%20summit%202025%20OD.pdf

[6] Sinha A, Mohapatra S, Sahoo KC, Mohanty S, Sahoo B, Pati S, et al. Motivation-opportunity-ability-behaviour of community members and program implementers towards mass drug administration for lymphatic filariasis elimination in India: a systematic review and implementation priority. Trans R Soc Trop Med Hyg. 2024;118(7):426–35. doi: 10.1093/trstmh/trae008 38506271

[7] Ahmad D, Mohanty I, Niyonsenga T. Improving birth preparedness and complication readiness in rural India through an integrated microfinance and health literacy programme: evidence from a quasi-experimental study. BMJ Open. 2022;12(2):e054318. doi: 10.1136/bmjopen-2021-054318 35190433 PMC8860014

[8] Mohanty S, Behera B, Sinha A, Bal M, Pati S, Sahoo PK. Mass drug administration to eliminate lymphatic filariasis: A population-based coverage and compliance study in Eastern India. Clinical Epidemiology and Global Health. 2024;30:101860. doi: 10.1016/j.cegh.2024.101860

[9] Mishra A, Kar K, Satapathy D. Mass drug administration against filariasis: a study on coverage and compliance in a coastal district of Odisha. Res J. 2015;47:13–8.

[10] Bhatia V, Giri PP, Sahoo SS, PS P, Sahu DP. Mass Drug Administration (MDA) for Elimination of Lymphatic Filariasis: Experiences from Nayagarh District of Odisha, India. Indian J Community Health. 2018;30(3):287–92. doi: 10.47203/ijch.2018.v30i03.017

[11] Ramaiah KD, Ottesen EA. Progress and impact of 13 years of the global programme to eliminate lymphatic filariasis on reducing the burden of filarial disease. PLoS Negl Trop Dis. 2014;8(11):e3319. doi: 10.1371/journal.pntd.0003319 25412180 PMC4239120

[12] Saeterdal I, Lewin S, Austvoll-Dahlgren A, Glenton C, Munabi-Babigumira S. Interventions aimed at communities to inform and/or educate about early childhood vaccination. Cochrane Database Syst Rev. 2014;2014(11):CD010232. doi: 10.1002/14651858.CD010232.pub2 25408540 PMC10880811

[13] Shepard DS, Lwin AK, Pulikkottil SI, Kalimuthu M, Arunachalam N, Tyagi BK, et al. Cost-effectiveness of vector control for supplementing mass drug administration for eliminating lymphatic filariasis in India. PLoS Negl Trop Dis. 2024;18(12):e0011835. doi: 10.1371/journal.pntd.0011835 39630851 PMC11649082

[14] Kanchan S, Gaidhane A. A study to assess the efficacy of print and digital health communication media tools (HCMT) in rural and urban communities. F1000Res. 2023;12:1314. doi: 10.12688/f1000research.139997.1 39380768 PMC11459119

[15] Ojo SO, Bailey DP, Brierley ML, Hewson DJ, Chater AM. Breaking barriers: using the behavior change wheel to develop a tailored intervention to overcome workplace inhibitors to breaking up sitting time. BMC Public Health. 2019;19(1):1126. doi: 10.1186/s12889-019-7468-8 31420033 PMC6697980

[16] Cane J, O’Connor D, Michie S. Validation of the theoretical domains framework for use in behaviour change and implementation research. Implement Sci. 2012;7:37. doi: 10.1186/1748-5908-7-37 22530986 PMC3483008

[17] Wang H, Blake H, Chattopadhyay K. Development of a School-Based Intervention to Increase Physical Activity Levels Among Chinese Children: A Systematic Iterative Process Based on Behavior Change Wheel and Theoretical Domains Framework. Front Public Health. 2021;9:610245. doi: 10.3389/fpubh.2021.610245 33987160 PMC8110714

[18] Meloncelli N, Wilkinson SA, Rushton A, Pateman K, Gallaher S, O’Connor H, et al. Co-designing The Healthy Gut Diet for Preventing Gestational Diabetes: Co-design methods and process outcomes. J Hum Nutr Diet. 2024;37(5):1231–44. doi: 10.1111/jhn.13341 38894634

[19] Mohapatra G. Evaluation of Mass Drug Administration Programme in Gajapati District, Odisha. JCD. 2018;50(03):11–4. doi: 10.24321/0019.5138.201814

[20] World Health Organization. Preventive chemotherapy: tools for improving the quality of reported data and information: a field manual for implementation. Geneva: World Health Organization. 2019. https://www.who.int/publications/i/item/9789241516464

[21] Michie S, van Stralen MM, West R. The behaviour change wheel: a new method for characterising and designing behaviour change interventions. Implement Sci. 2011;6:42. doi: 10.1186/1748-5908-6-42 21513547 PMC3096582

[22] Alexander KE, Brijnath B, Mazza D. Barriers and enablers to delivery of the Healthy Kids Check: an analysis informed by the Theoretical Domains Framework and COM-B model. Implement Sci. 2014;9:60. doi: 10.1186/1748-5908-9-60 24886520 PMC4047437

[23] Michie S, Atkins L, West R. The behaviour change wheel: a guide to designing interventions. London: Silverback Publishing. 2014.

[24] Michie S, Richardson M, Johnston M, Abraham C, Francis J, Hardeman W, et al. The behavior change technique taxonomy (v1) of 93 hierarchically clustered techniques: building an international consensus for the reporting of behavior change interventions. Ann Behav Med. 2013;46(1):81–95. doi: 10.1007/s12160-013-9486-6 23512568

[25] Landis JR, Koch GG. The measurement of observer agreement for categorical data. Biometrics. 1977;33(1):159–74. doi: 10.2307/2529310 843571

[26] Sinha A, et al. Facilitators and barriers in implementation of mass drug administration for the elimination of lymphatic filariasis: a qualitative study. PLoS Negl Trop Dis. 2022;16:e0010723. doi: 10.1371/journal.pntd.0010723

[27] Manyeh AK, et al. Evaluating context-specific evidence-based quality improvement interventions in Ghana: the case of lymphatic filariasis mass drug administration. PLoS Negl Trop Dis. 2021;15:e0009037. doi: 10.1371/journal.pntd.0009037 PMC7890643 33602322

[28] Krentel A, Damayanti R, Titaley CR, Suharno N, Bradley M, Lynam T. Improving Coverage and Compliance in Mass Drug Administration for the Elimination of LF in Two “Endgame” Districts in Indonesia Using Micronarrative Surveys. PLoS Negl Trop Dis. 2016;10(11):e0005027. doi: 10.1371/journal.pntd.0005027 27812107 PMC5094770

[29] Sinha A, Mohapatra S, Mohanty S, Pati S, Sahoo PK. Mass drug administration for lymphatic filariasis elimination amidst COVID-19 pandemic in Odisha, India: A step towards achieving SDG-3. Trop Doct. 2022;52(4):556–9. doi: 10.1177/00494755221098532 35770893 PMC9247629

[30] Bashar A, Nandekar SV, Shrivastava P, Singh RN, Srikrishnan P. Coverage and compliance of mass drug administration (MDA) programme for elimination of lymphatic filariasis in an endemic district of eastern Uttar Pradesh, India. J Vector Borne Dis. 2023;60(3):307–16. doi: 10.4103/0972-9062.374034 37843242

[31] Ramaiah KD, Das PK, Appavoo NC, Ramu K, Augustin DJ, Kumar KN, et al. A programme to eliminate lymphatic filariasis in Tamil Nadu state, India: compliance with annual single-dose DEC mass treatment and some related operational aspects. Trop Med Int Health. 2000;5(12):842–7. doi: 10.1046/j.1365-3156.2000.00659.x 11169272

[32] Kaur S, Kumar R, Lakshmi PVM, Kaur M. Effectiveness of a school-based behavioural change intervention in reducing chronic disease risk factors in Chandigarh, India: a cluster-randomised controlled trial. Lancet Reg Health Southeast Asia. 2024;21:100353. doi: 10.1016/j.lansea.2024.100353 38312946 PMC10832458

[33] Bhatia V, Singh AK, Giri PP, Sahoo DP. Effectiveness of a short-structured training programme on knowledge of healthcare providers and programme managers involved in maternal and child health programmes in Odisha, India: a quality improvement study. BMJ Open. 2021;11(8):e040841. doi: 10.1136/bmjopen-2020-040841 34404694 PMC8372814

[34] Maurer M, Mangrum R, Hilliard-Boone T, Amolegbe A, Carman KL, Forsythe L, et al. Understanding the Influence and Impact of Stakeholder Engagement in Patient-centered Outcomes Research: a Qualitative Study. J Gen Intern Med. 2022;37(Suppl 1):6–13. doi: 10.1007/s11606-021-07104-w 35349017 PMC8993962

[35] Mugo C, Njuguna I, Nduati M, Omondi V, Otieno V, Nyapara F, et al. From research to international scale-up: stakeholder engagement essential in successful design, evaluation and implementation of paediatric HIV testing intervention. Health Policy Plan. 2020;35(9):1180–7. doi: 10.1093/heapol/czaa089 32944754 PMC7810404

[36] Jagosh J, Macaulay AC, Pluye P, Salsberg J, Bush PL, Henderson J, et al. Uncovering the benefits of participatory research: implications of a realist review for health research and practice. Milbank Q. 2012;90(2):311–46. doi: 10.1111/j.1468-0009.2012.00665.x 22709390 PMC3460206

[37] Sales AE, Farr SL, Spertus JA. The Influence of Health Behavior Theory on Implementation Practice and Science: Brief Review and Commentary. Pharmacy (Basel). 2022;10(5):115. doi: 10.3390/pharmacy10050115 36136848 PMC9498588

[38] Cowdell F, Dyson J. How is the theoretical domains framework applied to developing health behaviour interventions? A systematic search and narrative synthesis. BMC Public Health. 2019;19(1):1180. doi: 10.1186/s12889-019-7442-5 31455327 PMC6712870

[39] Dyson J, Cowdell F. How is the Theoretical Domains Framework applied in designing interventions to support healthcare practitioner behaviour change? A systematic review. Int J Qual Health Care. 2021;33(3):mzab106. doi: 10.1093/intqhc/mzab106 34279637

