# Supplementary material for: Co-designing the ‘LEAD’ strategy: A theory-informed stakeholder-led behavioural intervention to improve mass drug administration compliance for lymphatic filariasis elimination in an urban block of Odisha, India
Source: PLoS Negl Trop Dis. 2026 Sep 16;20(9):e0014632. doi: 10.1371/journal.pntd.0014632 (PMC13645217; doi:10.1371/journal.pntd.0014632)
Supplement: Supplementary file 1 [file pntd.0014632.s003.pdf]

କ୍ରମାଂକ	ତାରିଖ	ସ୍ଥାନ / ଶ୍ରେଣୀ	କେଉଁମାନେ ଅଂଶଗ୍ରହଣ କଲେ?	ଅଂଶଗ୍ରହଣକାରୀ ଙ୍କ ସଂଖ୍ୟା	ମତାମତ (ଯଦି କିଛି ଅଛି)
1	___/ 03 / 25				
2	___/ 03 / 25				
3	___/ 03 / 25				
4	___/ 04 / 25				
5	___/ 04 / 25				
6	___/ 04 / 25				
7	___/ 04 / 25				
8	___/ 04 / 25				
9	___/ 04 / 25				
10	___/ 05 / 25				
11	___/ 05 / 25				
12	___/ 05 / 25				
13	___/ 05 / 25				
14	___/ 05 / 25				
15	___/ 05 / 25				
16	___/ 06 / 25				
17	___/ 06 / 25				
18	___/ 06 / 25				
19	___/ 06 / 25				
20	___/ 06 / 25				
21	___/ 06 / 25				
22	___/ 07 / 25				
23	___/ 07 / 25				
24	___/ 07 / 25				
25	___/ 07 / 25				
26	___/ 07 / 25				
27	___/ 07 / 25				
28	___/ 08 / 25				
29	___/ 08 / 25				
30	___/ 08 / 25				

ଫାଇଲେରିଆ ବା ବାତଜ୍ୱର ସ୍ୱାସ୍ଥ୍ୟ ଶିକ୍ଷା

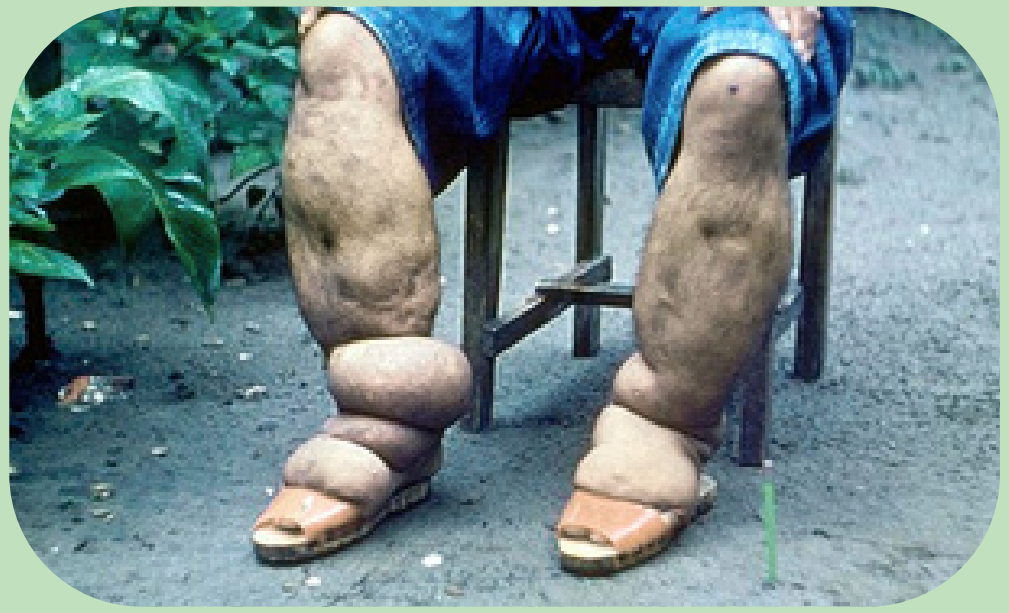

କିଛି ଜରୁରୀ ତଥ୍ୟ →

ନାମ: ____________________

ପଦବୀ____________________

ସ୍ଥାନ:____________________

ଆସନ୍ତୁ ସମସ୍ତେ ମିଳିମିଶି ଆମ ଅଞ୍ଚଳକୁ ଫାଇଲେରିଆ ମୁକ୍ତ କରିବା

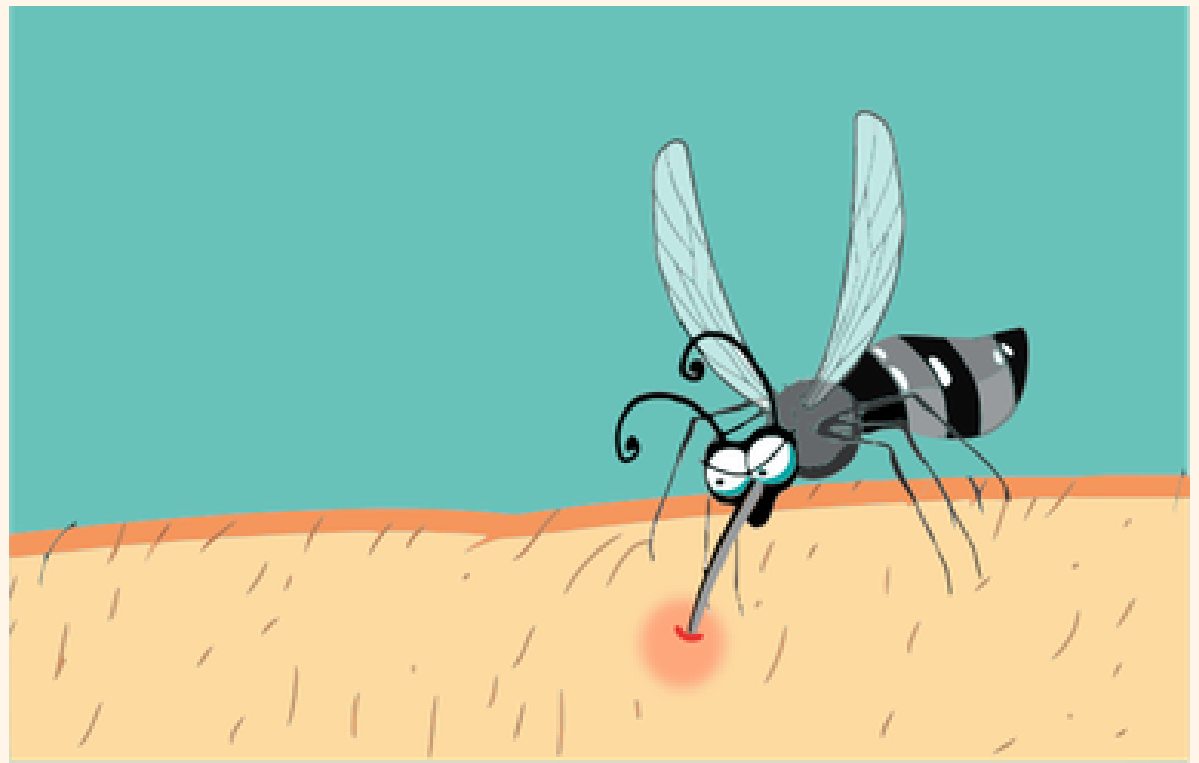

1 ଫାଇଲେରିଆ ରୋଗ କ'ଣ ଓ କିପରି ହୁଏ ?

- ଗୋଡ଼, ହାତ ଓ ପାଦ ଫୁଲି ଗୋଦର ହେଲେ ଆମେ ଫାଇଲେରିଆ/ ଗୋଦର ଗୋଡ ହୋଇଛି ବୋଲି କହିଥାଉ।
- ମଶା କାମୁଡ଼ିବା ଦ୍ୱାରା ଫାଇଲେରିଆ କୀଟାଣୁ ସଂକ୍ରମିତ ବ୍ୟକ୍ତିଙ୍କଠୁ ଅନ୍ୟ ଜଣେ ବ୍ୟକ୍ତିଙ୍କ ଶରୀରରେ ପ୍ରବେଶ କରିଥାଏ ।
- ଏହି ରୋଗ ବଂଶାନୁଗତ ନୁହେଁ । ମଶା କାମୁଡ଼ିବା ଦ୍ୱାରା ବ୍ୟାପି ଥାଏ ।

2 ଫାଇଲେରିଆ ରୋଗ ର ଲକ୍ଷଣ କ'ଣ ?

- ପ୍ରାରମ୍ଭିକ ଲକ୍ଷଣମାନେ ହେଉଛି ଜ୍ୱର ଆସିବା, ଦେହ ହାତ ଘୋଳା ବିନ୍ଧା ହେବା, ଚର୍ମରେ ଲାଲ ଦାଗ ପଡ଼ିବା ଓ ରକ୍ତ ପଡ଼ିବା ।
- ପର୍ଯ୍ୟାୟକ୍ରମେ ଗୋଡ଼ ଫୁଲିବା, ହାତ ଫୁଲିବା, ସ୍ତନ ଫୁଲିବା, ଅଣ୍ଡକୋଷ ଫୁଲିବା ଓ ଗୋଦର ଗୋଡ଼ ହେବା ଲକ୍ଷଣ ଦେଖାଯାଏ। ପ୍ରଥମ ଅବସ୍ଥାରୁ ଉପଯୁକ୍ତ ଯତ୍ନ ନ ନେଲେ ଫୁଲା କମେ ନାହିଁ ଓ ଅଙ୍ଗଟି ବିକଳାଙ୍ଗ ହୋଇଯାଏ ।

3 ଫାଇଲେରିଆ କୀଟାଣୁ ନଷ୍ଟ କରାଯିବ କିପରି ?

- ଏଥିପାଇଁ "ସାର୍ବଜନୀନ ଔଷଧ ସେବନ" / **Mass Drug Administration (MDA)** କାର୍ଯ୍ୟକ୍ରମକୁ ଫାଇଲେରିଆ ପ୍ରବଣ ଜିଲ୍ଲାମାନଙ୍କରେ କାର୍ଯ୍ୟକାରୀ କରାଯାଉଛି। ଏଥିରେ ସମସ୍ତଙ୍କୁ ଡ଼ିଇସି + ଆଲ୍‌ବେଣ୍ଡାଜୋଲ୍ ଔଷଧ ଦିଆଯାଏ।
- ଲଗାତାର ୫ ରୁ ୬ ବର୍ଷ ପର୍ଯ୍ୟନ୍ତ ଯଦି ଗୋଟିଏ ଗୋଷ୍ଠୀରେ ଶତକଡ଼ା ୮୫ ଭାଗରୁ ଅଧିକ ଲୋକ ବର୍ଷର ଗୋଟିଏ ଦିନ ଔଷଧ ସେବନ କରନ୍ତି, ତେବେ ଗୋଷ୍ଠୀରୁ ଫାଇଲେରିଆ ନିରାକରଣ ହୋଇପାରିବ।
- ମଶା ବଂଶ ବୃଦ୍ଧି ରୋକିବା ଓ ମଶା କାମୁଡ଼ିବା ରୋକିବା ପାଇଁ ଏହି ଉପାୟମାନେ ଅବଲମ୍ବନ କରନ୍ତୁ:
  - ଘର ଆଖପାଖରେ ପାଣି ଜମିବାକୁ ଦେବେନାହିଁ।
  - ନାଳ ଓ ନର୍ଦ୍ଦମାକୁ ଢାଙ୍କି କରି ରଖିବେ।
  - ସବୁବେଳେ ମଶାରି ଟାଙ୍ଗି ଶୋଇବେ।
  - ମଶା ଘଉଡ଼ାଇବା ପାଇଁ ମଶାଧୂପ ବ୍ୟବହାର କରିବେ।

4 କେଉଁମାନେ ଏହି ଔଷଧ ସେବନ କରିବେ ?

- ୨ ରୁ ଅଧିକ ବୟସର ସମସ୍ତ ଯୋଗ୍ୟ ବ୍ୟକ୍ତି ଏହି ଔଷଧ ସେବନ କରି ପାରିବେ।
- ୨ ରୁ କମ୍ ବର୍ଷର ଶିଶୁ, ଗର୍ଭବତୀ ମହିଳା ଏବଂ ଗୁରତର ଅସୁସ୍ଥ ହିତାଧିକାରୀ ଙ୍କୁ ଏମତିଏ ଔଷଧ ଦିଆଯିବ ନାହିଁ ।

5 କୌଣସି ଲକ୍ଷଣ ନ ଥିଲେ ମଧ୍ୟ ଜଣେ କାହିଁକି ଔଷଧ ଖାଇବେ ?

- ଫାଇଲେରିଆ କୀଟାଣୁ ଶରୀରରେ ପ୍ରବେଶ କରିବାର ୧୦ ରୁ ୧୫ ବର୍ଷ ପରେ ରୋଗର ଲକ୍ଷଣମାନ ଦେଖାଯାଇଥାଏ ।
- ଲକ୍ଷଣ ଦେଖାନଗଲେ ମଧ୍ୟ ଆମ ଅଞ୍ଚଳରେ ରୋଗୀ ଓ ସଂକ୍ରମିତ ମଶା ଥିବାରୁ ଏହା ସଂକ୍ରମିତ ହେବାର ସମ୍ଭାବନା ଅଛି। ଏଥିପାଇଁ ଫାଇଲେରିଆ ଔଷଧ ଖାଇବା ନିହାତି ଆବଶ୍ୟକ।

6 ଔଷଧ ଖାଇବା ପରେ କୌଣସି ପାର୍ଶ୍ୱ ପ୍ରତିକ୍ରିୟା ଦେଖାଯାଏ କି ?

- ପାର୍ଶ୍ୱପ୍ରତିକ୍ରିୟା ସମସ୍ତଙ୍କ କ୍ଷେତ୍ରରେ ଦେଖାଯାଏ ନାହିଁ ।
- ଯେଉଁମାନଙ୍କ ଦେହରେ ଅଧିକ ପରିମାଣର ଫାଇଲେରିଆ କୀଟାଣୁ ଥାଆନ୍ତି, ସେମାନଙ୍କଠାରେ କେତେକ ସାଧାରଣ ପାର୍ଶ୍ୱପ୍ରତିକ୍ରିୟା ଦେଖାଯାଇପାରେ। ଯଥା: ଜ୍ୱର, ମୁଣ୍ଡବିନ୍ଧା, ଦେହହାତ ବ୍ୟଥା, ମୁଣ୍ଡ ବୁଲେଇବା, ଚର୍ମରେ ଲାଲ ହୋଇ ଫଳିଯିବା ଓ ବେଳେ ବେଳେ ଚର୍ମ କୁଣ୍ଡାଇ ହେବା।
- ଏ ସବୁ ପାର୍ଶ୍ୱପ୍ରତିକ୍ରିୟା ୨୪ ଘଣ୍ଟା ମଧ୍ୟରେ ଆପେ ଆପେ ଭଲ ହୋଇଯାଏ । ଯଦି ପାର୍ଶ୍ୱ ପ୍ରତିକ୍ରିୟା ଲାଗିରହେ, ତେବେ ପାଖ ସ୍ୱାସ୍ଥ୍ୟକେନ୍ଦ୍ରକୁ ରୋଗୀକୁ ନେଇ ଚିକିତ୍ସା କରାଯାଇପାରିବ।

ଦୟାକରି ସମସ୍ତଙ୍କୁ ଏହି ସନ୍ଦେଶ ପ୍ରଚାର କରିବାକୁ କହନ୍ତୁ
