# Supplementary material for: Co-designing the ‘LEAD’ strategy: A theory-informed stakeholder-led behavioural intervention to improve mass drug administration compliance for lymphatic filariasis elimination in an urban block of Odisha, India
Source: PLoS Negl Trop Dis. 2026 Sep 16;20(9):e0014632. doi: 10.1371/journal.pntd.0014632 (PMC13645217; doi:10.1371/journal.pntd.0014632)
Supplement: Supplementary file 2 [file pntd.0014632.s001.docx]

| **TDF-Based Qualitative Instrument for Exploring Barriers and Enablers of**  **MDA Compliance and LF Intervention Implementation**  **(Estimated time of the interview 30min – 1hr)**  **Semi-structured interviews**  *(These are only guides to prompt the interviewer and interviewee to adhere to the topic. Please feel free to add the themes and concerns as you see fit)*  **Introduction:**  I am ………………………… from the ………………………and I am approaching you to conduct a study related to the **implementation of the Lymphatic Filariasis (LF) elimination program and Mass Drug Administration (MDA) activities** in this area. As you know, LF continues to be a public health concern, and achieving high MDA coverage and compliance is critical for elimination. I would like to know your perspectives and experiences regarding health education, drug distribution, and barriers/enablers in MDA implementation.  There are no consequences attached to participating in this study. In addition, if you agree, I would like to audio-record this interview for transcription and analysis. I would like to inform you that this interview will be anonymized, the information you provide will be completely confidential, and the storage of all data including recordings will be done on a secure server of our institution with restricted access to the study team. You are free to drop out of the interview at any time, and you do not need to provide any reason for doing so.  If you agree, I would like to begin by asking you some questions regarding your role in LF/MDA activities.  **AUDIO FILE NAME:** ______________________ | | |
| --- | --- | --- |
| **SECTION-I: Demographic and role characteristics** | | |
| **Socio-demographic characteristics of respondents** | Age |  |
|  | Gender |  |
|  | Region (district/block of their role) |  |
|  | Municipality/ward/village (of their role) |  |
|  | Institution/organization (e.g., Health Department, School, Anganwadi Centre, Community Organization) |  |
|  | Type of institution (public, government, NGO, community-based) |  |
|  | Position/designation (e.g., ASHA, ANM, AWW, CDD, School Teacher, Ward Counsellors, Program Officer, Supervisor) |  |
|  | Years of experience in this role |  |
|  | If not currently in this role, total period of experience in the role |  |
|  | Education level |  |
| **SECTION-II: Prompts for FLHWs (ASHAs, ANMs, AWWs, CDDs)** | | |
| **Themes / Domains** | **Guide** | |
| General Questions | Can you describe your current role in the community?  Have you participated in any activities related to lymphatic filariasis or MDA? | |
| Knowledge | What do you know about Lymphatic Filariasis (LF)?  What information do you provide to others about LF and MDA?  PROBE: What causes LF? How is it prevented? | |
| Skills | Do you feel confident delivering sessions on LF and MDA?  Have you received any training on how to conduct health education?  PROBE: Was the training helpful? Do you need more support? | |
| Beliefs About Capabilities | Do you believe you can effectively motivate others to take MDA drugs?  What helps or limits your confidence? | |
| Beliefs About Consequences | What do people think will happen if they take MDA drugs?  Are there any side effects or risks that people fear?  PROBE: Have you heard of anyone refusing drugs because of fear? | |
| Social/Professional Role and Identity | Do you consider it part of your role to promote LF/MDA?  How do others (peers/community) view your involvement in these activities? | |
| Social Influences | Who supports you in your efforts?  Are there leaders or influential people who help spread the message?  PROBE: Are you more likely to act if your peers or supervisors encourage you? | |
| Environmental Context and Resources | Do you have enough time and materials to conduct the sessions?  Are there any issues with location, scheduling, or holidays?  PROBE: Do WhatsApp groups function well for sharing messages? | |
| Behavioural Regulation | How do you remember or plan your LF education activities?  Do you maintain any log, checklist, or follow-up plan?  PROBE: Do you get reminders or updates? | |
| Emotion | How do you feel when educating others about LF or promoting MDA?  Have there been emotional challenges (e.g., rejection, anxiety, fear)? | |
| Closing Questions | What are the biggest challenges you face while implementing LF/MDA awareness?  What could be done to support you better?  Any suggestions for improving LF-related health education? | |

| **SECTION-III: Prompts for Ward Counsellors, MDA Recipients/Non-Recipients, and School Teachers** | |
| --- | --- |
| **Themes / Domains** | **Guide** |
| General Questions (For teachers) | Can you describe your current role in the school?  Have you participated in any activities related to lymphatic filariasis or MDA? |
| Knowledge | What do you know about lymphatic filariasis and how it spreads?  What do you know about the MDA program in your area?  Have you received any information, training, or materials on LF or MDA?  What role do you think community leaders, teachers, or local health workers have in promoting MDA? |
| Skills | How comfortable do you feel in talking to others (students, families, or community members) about LF and MDA?  Have you used or seen any tools (e.g., posters, meetings, brochures, videos) to share LF-related messages?  Are there skills or support you think you need to communicate about MDA more effectively? |
| Beliefs About Capabilities | Do you feel confident that you can encourage families and communities to participate in MDA?  What makes it easier or harder for you to do so? |
| Beliefs About Consequences | What do you think happens if people do not take the MDA drugs?  What might happen if leaders, teachers, or community members do not talk about LF and MDA? |
| Motivation and Intentions | How motivated are you to support the MDA program in your community/school?  Would you be willing to integrate MDA promotion into your routine activities (e.g., school assemblies, ward meetings, community gatherings)? |
| Social/Professional Role and Identity | Do you see yourself (as a ward counsellor/community member/teacher) as someone who should spread awareness about MDA? Why or why not?  How do others in your role (colleagues, neighbours, peers) view their responsibility in MDA promotion? |
| Social Influences | How do families, students, or community members respond when you talk about MDA?  Do you think children or community leaders can influence families to take the drugs?  Are there any influential figures (local leaders, doctors, religious figures) who strongly support or discourage MDA? |
| Environmental Context and Resources | What support do you get from health workers, schools, or local authorities for promoting MDA?  Are there challenges (e.g., time, materials, competing responsibilities) that make it difficult to carry out MDA awareness activities? |
| Behavioural Regulation | Have you developed any strategies (reminders, group efforts, coordination with health workers) to support MDA promotion?  What kind of support (e.g., reminders, materials, recognition) would help you stay consistent in this effort? |
| Emotion | How do you feel when asked to support health programs like MDA, in addition to your usual responsibilities?  Have you faced any resistance, discomfort, or challenges when trying to promote MDA? |
| Closing Questions | What would make it easier for people in your community to participate in MDA?  Do you have any other suggestions or experiences you would like to share? |

| **SECTION-IV: Prompts for State-Level Program Officers & MDA Supervisors** | |
| --- | --- |
| **Themes / Domains** | **Guide** |
| General Questions | Can you describe your current role?  Have you participated in any activities related to lymphatic filariasis or MDA? |
| Knowledge  (Awareness of LF, MDA processes, and current implementation strategies) | What is your understanding of the key drivers of low MDA compliance in endemic areas?  Are program officers and supervisors generally trained on behavior change communication strategies?  How familiar are you with national guidelines on LF elimination and health education delivery? |
| Skills  (Ability to support, train, or monitor MDA delivery and health education) | What specific skills are needed by MDA supervisors and FLHWs to deliver effective LF messaging?  Do current training programs address those skill gaps adequately?  Are there challenges in training or upskilling field teams? |
| Beliefs About Capabilities  (Confidence in the system’s or individuals’ ability to influence MDA outcomes) | How confident are you that the existing delivery structure can achieve high MDA compliance?  What constraints reduce the system’s capacity to deliver effective LF education or mobilization? |
| Beliefs About Consequences  (Perceptions about the impact of strategies or failures in implementation) | What are the consequences of not addressing behavioral barriers (e.g., fear of side effects, low awareness) in MDA campaigns?  How impactful do you think health education sessions are on MDA compliance? |
| Motivation and Intentions  (Commitment to program goals and willingness to adapt strategies) | How motivated is the current system to innovate or adapt behavior change strategies to local contexts?  Are there any plans to revise or improve MDA communication approaches? |
| Social Influences  (Influence of political actors, partners, and community stakeholders) | How does engagement with local leaders, NGOs, or SHG members affect MDA implementation?  What kind of partnerships or support systems have proven most effective? |
| Environmental Context and Resources  (Infrastructure, logistics, and systemic constraints or enablers) | What are the main logistical or operational challenges in organizing MDA and related IEC/BCC efforts?  Are there adequate resources (staff, IEC materials, monitoring tools) for successful implementation? |
| Behavioural Regulation  (Systems or strategies used to monitor and improve program delivery) | How is MDA performance currently monitored at your level?  Are there structured feedback loops between frontline staff and supervisors?  What improvements are needed in terms of tracking health education sessions or community mobilization? |
| Emotion  (Emotional investment or fatigue in program oversight) | How do you personally feel about overseeing LF elimination efforts in your area?  Do you or your teams experience burnout or discouragement during MDA rounds? |
| Closing Questions | In your opinion, what would a more effective behavior change strategy for LF elimination look like in Odisha?  Are there any additional suggestions or success stories you would like to share? |

The End
