# Supplementary material for: Co-designing the ‘LEAD’ strategy: A theory-informed stakeholder-led behavioural intervention to improve mass drug administration compliance for lymphatic filariasis elimination in an urban block of Odisha, India
Source: PLoS Negl Trop Dis. 2026 Sep 16;20(9):e0014632. doi: 10.1371/journal.pntd.0014632 (PMC13645217; doi:10.1371/journal.pntd.0014632)
Supplement: Supplementary file 3 [file pntd.0014632.s002.docx]

| **No** | **Item** | **Guide questions/description** | **Answer-/ position in article** |
| --- | --- | --- | --- |
| **Domain 1: Research team and reflexivity** | | |  |
| *Personal Characteristics* | | |  |
| 1. | Interviewer/facilitator | Which author/s conducted the interview or focus group? | See Methods – Data management and analysis |
| 2.  3.  4.  5. | Credentials Occupation Gender  Experience and training | What were the researcher's credentials? *E.g. PhD, MD* What was their occupation at the time of the study?  Was the researcher male or female?  What experience or training did the researcher have? |  |
| **Answers on criteria 2-5**. Authors:  Dr Tanveer Rehman, MBBS, MD – Scientist C(Medical) - extensive experience in clinical practice, research and public health. (Male)  Ms Ananya Anurakta Pattanaik, B.Sc., MPH – Project Research Scientist-I, experience in public health research (Both qualitative and quantitative research) (Female)  Dr Madhusmita Bal, M.Sc, M.Phil, Ph.D. – Scientist-E, with experience in **longitudinal studies in filariasis-endemic populations** and **immunological evaluation.** (Female)  Dr Prakash Kumar Sahoo, M. Sc., Ph.D. – Scientist-D, with research experience in the field of Immunology, Biochemistry, Molecular Biology and virology of infectious diseases/vector-borne diseases (Male)  Dr Krushna Chandra Sahoo, PhD (Public Health, Karolinska Institute, Sweden) – Scientist-D, Health Technology Assessment in India (HTAIn), extensive experience in qualitative research and health behavior (Male)  Dr Shubhashisha Mohanty, MBBS, MD – Joint Director of Health Services, National Vector Borne Disease Control Programme, Department of Health and Family Welfare, Government of Odisha, with significant experience in **public health program implementation and management,** particularly in **vector-borne disease control programs** at the state level. (Male)  Dr Sandipana Pati, MBBS, Ph.D. – Joint Director at State Institute of Health and Family Welfare with expertise in **health systems, training, and public health program strengthening.** (Female)  Dr Sanghamitra Pati, MBBS, MD, MPH – Additional Director General at the Indian Council of Medical Research (ICMR) in New Delhi with extensive training and leadership experience in clinical research, epidemiology, and public health policy, guiding large-scale national research initiatives. (Female) | | | See box on the left. |
| Interviewers:   - Ms Ananya Anurakta Pattanaik – female, Project Research Scientist-I - Dr Tanveer Rehman – male, Scientist-C (Medical) | | |  |
| *Relationship with participants* | | |  |
| 6. | Relationship established | Was a relationship established prior to study  commencement? | No prior relationship existed between the researchers and participants before study commencement. Participants were approached through local healthcare providers and community volunteers with whom they had an existing working relationship. |
| 7. | Participant knowledge of the interviewer | What did the participants know about the researcher? e*.g. personal goals, reasons for doing the research* | Prior to each session, participants were informed that the researchers were public health scientists from ICMR-Regional Medical Research Centre, Bhubaneswar, conducting a formative study on MDA compliance for lymphatic filariasis elimination. The study objectives, voluntary nature of participation, and confidentiality of responses were explained during the informed consent process. |
| 8. | Interviewer characteristics | What characteristics were reported about the interviewer/facilitator? e.g. *Bias, assumptions, reasons and*  *interests in the research topic* | The lead interviewers (Dr Tanveer Rehman and Ms Ananya Anurakta Pattanaik) had no supervisory or programmatic authority over participants, minimising power imbalance. Both researchers were aware of their positionality as public health scientists with an institutional interest in LF elimination, and reflexivity was maintained throughout data collection and analysis. No prior assumptions regarding specific barriers were imposed on participants. |
|  |  |  |  |
| **Domain 2: study design** | | |  |
| *Theoretical framework* | | |  |
| 9. | Methodological orientation and Theory | What methodological orientation was stated to underpin the study? *e.g. grounded theory, discourse analysis, ethnography, phenomenology, content analysis* | Thematic framework analysis guided by a deductive approach using the Theoretical Domains Framework (TDF) and the COM-B model. See Methods – Theoretical framework and Data management and analysis sections. |
| *Participant selection* | | |  |
| 10. | Sampling | How were participants selected? *e.g. purposive, convenience,*  *consecutive, snowball* | Purposive maximum variation sampling was used to capture diverse perspectives across three stakeholder categories: MDA implementers (ASHAs, AWWs, ANMs, CDDs), community actors (community members, schoolteachers, ward councillors), and programme supervisors (district NVBDCP officers). See Methods – Qualitative interview section. |
| 11. | Method of approach | How were participants approached? e*.g. face-to-face,*  *telephone, mail, email* | Participants were approached face-to-face with the support of local healthcare providers and community volunteers. Recruitment was facilitated by the district health office and community liaisons.  See Methods – Qualitative interview section. |
| 12. | Sample size | How many participants were in the study? | 20 participants in total: FGD 1 – 9 FLHWs/CDDs; FGD 2 – 8 community members; KII – 3 district officials/MDA supervisors. See Table 2 (participant characteristics). |
| 13. | Non-participation | How many people refused to participate or dropped out? Reasons? | All participants who were approached and consented agreed to participate. There were no refusals or dropouts during the qualitative data collection. Participants were recruited purposively and had confirmed availability prior to sessions. |
| *Setting* | | |  |
| 14. | Setting of data collection | Where was the data collected? e*.g. home, clinic, workplace* | FGDs were conducted at a local community meeting space within Athagarh NAC, ensuring privacy and comfort. KIIs were held at the respective offices of the district NVBDCP officials and the MDA supervisor. All settings were familiar to participants and free from interruptions.  See Methods – Data management and analysis section. |
| 15. | Presence of non-  participants | Was anyone else present besides the participants and  researchers? | No non-participants were present during any FGD or KII sessions. Sessions were conducted in private settings with only the participants and the research team (facilitator and note-taker) present. |
| 16. | Description of sample | What are the important characteristics of the sample? *e.g.*  *demographic data, date* | Socio-demographic characteristics of all qualitative participants (age, gender, education, occupation, work experience) are presented in Table 2 of the manuscript. FGD 1 comprised 9 FLHWs/CDDs (78% female; median age 37 years). FGD 2 comprised 8 community members (50% female; median age 45 years). KII comprised 3 programme officials (33% female; median age 43 years). |
| *Data collection* | | |  |

| 17. | Interview guide | Were questions, prompts, guides provided by the authors? Was it pilot tested? | Yes. A semi-structured FGD and KII guide was developed by the research team using open-ended questions and probes aligned to the 14 domains of the TDF. The face validity of the guide was reviewed independently by three authors with prior experience in TDF-based behavioural studies. The guide was not formally pilot tested with a separate group but was iteratively refined based on team review. See S1 File (supplementary) and Methods – Qualitative interview section. |
| --- | --- | --- | --- |
| 18. | Repeat interviews | Were repeat interviews carried out? If yes, how many? | No. Repeat interviews were not conducted. Each participant was interviewed or participated in a FGD once only. |
| 19. | Audio/visual recording | Did the research use audio or visual recording to collect the  data? | Yes. All FGD and KII sessions were audio recorded following written informed consent from participants. No visual (video) recording was used. See Methods – Data management and analysis section. |
| 20. | Field notes | Were field notes made during and/or after the interview or focus group? | Yes. Field notes were made by the co-facilitator during and immediately after each FGD and KII session to capture non-verbal cues, group dynamics, and contextual observations that could not be captured by audio recording alone. These were used to complement transcript analysis.  See Methods – Data management and analysis section. |
| 21. | Duration | What was the duration of the interviews or focus group? | Each FGD and KII session lasted approximately 60–90 minutes. See Methods – Data management and analysis section. |
| 22. | Data saturation | Was data saturation discussed? | Data saturation was assessed iteratively during the coding process. Given the geographically defined and socially homogeneous study area (Athagarh NAC) and the limited stakeholder pool, no new themes emerged after the second FGD and third KII, indicating adequate saturation within this context. The purposive maximum variation sampling further ensured breadth of perspectives. |
| 23. | Transcripts returned | Were transcripts returned to participants for comment and/or correction? | Transcripts were not returned to individual participants for review, as this was not operationally feasible given the community-based setting and participant availability. However, synthesised findings were presented to stakeholders during the first co-design workshop, where participants validated the behavioural diagnosis and confirmed the accuracy of mapped themes, serving as a form of member checking. |
|  |  |  |  |
| **Domain 3: analysis and findings** | | |  |
| *Data analysis* | | |  |
| 24. | Number of data coders | How many data coders coded the data? | Two trained researchers independently coded all transcripts. Inter-coder reliability was assessed using Cohen's kappa (κ = 0.86; 95% CI: 0.83–0.89), indicating high agreement. Discrepancies were resolved through consensus, with arbitration by a third qualitative expert when necessary. See Methods – Data management and analysis section. |
| 25. | Description of the coding  tree | Did authors provide a description of the coding tree? | Yes. A deductive coding framework was developed based on the 14 domains of the TDF. Each transcript segment was coded to one or more TDF domain nodes, with sub-nodes distinguishing barriers and facilitators. Coded segments were subsequently mapped to COM-B components. The coding structure is reflected in Table 4 of the manuscript. See Methods – Data management and analysis section. |
| 26. | Derivation of themes | Were themes identified in advance or derived from the  data? | Themes were identified deductively, using the 14 domains of the TDF as a pre-specified coding framework. This approach was informed by the study's theoretical underpinning (TDF and COM-B model) rather than allowing themes to emerge inductively from the data. See Methods – Data management and analysis section. |
| 27. | Software | What software, if applicable, was used to manage the data? | NVivo 12 (QSR International) was used for data management, organisation, and coding of qualitative transcripts. See Methods – Data management and analysis section. |
| 28. | Participant checking | Did participants provide feedback on the findings? | Individual participant checking of transcripts was not conducted. However, synthesised findings and the behavioural diagnosis were presented to stakeholders during the first co-design workshop. Participants reviewed, validated, and where necessary corrected the mapped TDF domains and COM-B components, constituting a group-level member checking process. See Methods – Stakeholder Engagement and Intervention Co-Design section. |
| *Reporting* | | |  |
| 29. | Quotations presented | Were participant quotations presented to illustrate the themes / findings? Was each quotation identified? e*.g.*  *participant number* | Yes. Verbatim participant quotations are presented in supplementary file 4 of the manuscript to illustrate each of the ten prioritised TDF domains. Each quotation is identified by a participant code (e.g., A1 for ASHA, T1 for teacher, S1 for MDA supervisor, O1 for NVBDCP official, N1 for ANM, W1 for Anganwadi worker, V1 for community volunteer, C1 for ward councillor, R1 for MDA recipient). See Results section. |
| 30. | Data and findings  consistent | Was there consistency between the data presented and the  findings? | Yes. Qualitative findings are presented alongside quantitative evidence from the KAP survey in Table 4, enabling cross-validation. Both data sources are consistently mapped to the same TDF domains and COM-B components, demonstrating coherence between the data presented and the conclusions drawn. See Results section. |
| 31. | Clarity of major themes | Were major themes clearly presented in the findings? | Yes. Ten prioritised TDF domains were identified as major themes and are clearly presented with corresponding COM-B component mappings, quantitative evidence, and illustrative qualitative quotes in supplementary file 4. The qualitative insights section of the Results also provides a narrative summary of major themes across community, implementer, and health system levels. See Results section. |
| 32. | Clarity of minor themes | Is there a description of diverse cases or discussion of minor  themes? | Yes. Both barriers and facilitators are presented for each TDF domain in supplementary file 4, ensuring that positive enabling factors (minor themes) are reported alongside the dominant barrier themes. The facilitators identified (e.g., trusted community messengers, existing platforms, peer influence) are discussed in the Results and inform the LEAD intervention design. See Results and Discussion sections. |
