# Supplementary material for: Co-designing the ‘LEAD’ strategy: A theory-informed stakeholder-led behavioural intervention to improve mass drug administration compliance for lymphatic filariasis elimination in an urban block of Odisha, India
Source: PLoS Negl Trop Dis. 2026 Sep 16;20(9):e0014632. doi: 10.1371/journal.pntd.0014632 (PMC13645217; doi:10.1371/journal.pntd.0014632)
Supplement: Supplementary file 4 [file pntd.0014632.s004.docx]

| **Illustrative Quotes**  **Supplementary file 4: Table 4(Extended version). Mapping of illustrative quotes from focus group discussions, aligned with quantitative survey findings, to the Theoretical Domains Framework (TDF) and COM-B model** | **Quantitative Evidence** | **Determinants** | **Domain**  **(Definition)*** | **COM-B System** |
| --- | --- | --- | --- | --- |
| *"Most people don’t even know what filariasis is. They ask us, ‘Why are you giving this medicine?’” (A3)*  *“Others think that because the program is repeated, it means it didn’t work the last time.”(A1)* | In the community, 33% of respondents were unaware of LF causes; 22% mistakenly believed it to be hereditary. | Lack of community knowledge about LF (Barrier) | **1. Knowledge**  (An awareness of the existence of something) | **Psychological Capability** |
| *"If we had started awareness sessions at least 2–3 months before, it would’ve made a big difference."(A2)* |  | Need for early education (Facilitator) |  |  |
| *“Parents don’t have much knowledge about LF or the MDA program.”(T2)* |  | Parental knowledge gap (Barrier) |  |  |
| *"Some people ask very tricky questions — like, 'Will this tablet interact with my BP medicine?' or 'How do you know what’s inside my body?'"(A3)* | Fear of drug interactions was a barrier among 8% of non-compliant individuals, primarily those with pre-existing conditions (hypertension, diabetes, or tuberculosis). | Difficulty handling skepticism under time pressure (Barrier) | **2. Skills**  (An ability or proficiency acquired through practice) | **Psychological Capability** |
| *"Sometimes I tell people, 'Look, I took it myself. Nothing happened.' That helps a lot."(V1)* |  | Using personal testimony (Facilitator) |  |  |
| *"Yes, if we have time and support. People listen to us, especially when we explain calmly."(V2)* | A majority of respondents (72%, n = 289) reported that Community Drug Distributors (CDDs) effectively clarified their doubts regarding Mass Drug Administration (MDA). | Confidence in community influence (Facilitator) | **3. Beliefs About Capabilities**  (Acceptance of the truth, reality, or validity about an ability, talent, or facility that a person can put to constructive use) | **Reflective Motivation** |
| *"If children come home and say 'Mummy, teacher told us to take this medicine,' it changes the parent’s mind."(N1)* |  | Leveraging peer influence (Facilitator) |  |  |
| *"Through poster-making competitions, students understand better and feel involved."(T2)* |  | Creative pedagogy confidence (Facilitator) |  |  |
| *"So many fears! Vomiting, stomach pain, dizziness… even if one child faints in school after taking MDA, the whole basti gets scared."(A1)* | The main reason for MDA non-consumption was fear of side effects (40%). | Fear of side effects (Barrier) | **4. Beliefs About Consequences**  (Acceptance of the truth, reality, or validity about outcomes of a behaviour in a given situation) | **Reflective Motivation** |
| *"One neighbour fainted once after taking tablets. Now half the lane refuses every year."(R3)* |  |  |  |  |
| *“…..So when we ask for consent or try to give tablets at school, many parents simply say no. They are scared of side effects “(T2)* |  | Refusal due to side effect fears (Barrier) |  |  |
| *"We already did awareness during the MDA campaign. I don’t think it’s necessary again." (T1)*  *"I didn’t think it was our responsibility to explain all this." (T2)* |  | Limited teacher role awareness (Barrier) | **5**.**Social/Professional Role & Identity**  (A coherent set of behaviours and displayed personal qualities of an individual in a social or work setting) | **Reflective Motivation** |
| *"SHG didis can be very helpful. Women listen to them. If they say 'Take the medicine,' people will take it."(W1)* |  | Trusted community messengers (Facilitator) | **6. Social Influences**  (Those interpersonal processes that can cause individuals to change their thoughts, feelings, or behaviours) | **Social Opportunity** |
| *“The local Anganwadi didi and ASHA didis convinced many people to take MDA.” (M1)* |  | Influence of local trusted figures (Facilitator) |  |  |
| *"Youth club boys help talk to men, especially in community meetings. As it is difficult to convince men than women for us "(C1)* | Female compliance (n=128, 56 %) was higher than male compliance (n=101, 44%). | Youth engagement (Facilitator) |  |  |
| *"Students might convince parents if taught regularly."(T2)* |  | Student-to-parent influence (Facilitator) |  |  |
| *"People were not at home. Some work in fields, some go to town."(A1)* | 13.3% of community members reported missing tablets due to absence during MDA. | Physical access challenges (Barrier) | **7. Environmental Context & Resources**  (Any circumstance of a person's situation or environment that discourages or encourages the development of skills and abilities, independence, social competence, and adaptive behaviour) | **Physical Opportunity** |
| *“Polio got more publicity and focus earlier, with big drives. LF doesn’t get that kind of attention.”(O1)* |  | Lack of visibility compared to other programs (Barrier) |  |  |
| *"We should use every platform — UHSND, Kishori Baithak, SHG meetings. It doesn’t need extra effort." (O2)* | 57% of participants heard about MDA through UHSND events, community gatherings, or health workers. | Existing platforms for outreach (Facilitator) |  |  |
| *“We’re overloaded. Every month we have immunization, nutrition day, ANC check-ups, sometimes even two programs in one day. We may forget to do health education sessions "(A2)* |  | Need a record system to observe LF HE sessions (Barrier) | **8. Behavioural Regulation**  (Anything aimed at managing or changing objectively observed or measured actions) | **Psychological Capability/ Physical Opportunity** |
| *"Posters, audio messages, videos help. Not everyone is home, but they see digital content."(C2)* | 78% (n = 312) of respondents reported that they had not received any health education content related to Lymphatic Filariasis through social media or mass media. | Multi-channel reminders (Facilitator) |  |  |
| *“We have a parent-teacher group. We can definitely share some messages with parents through that group. Maybe once a week.” (T3)* |  | Parent-teacher group utility (Facilitator) |  |  |
| *"Mostly educated people say, 'Who are you to tell me? I know more than you’- it hurts."(A3)* | Compliance was highest among those with no formal education (75%) and lowest among the highly educated (44%). | Community resistance (Barrier) | **9. Emotion**  (A complex reaction pattern, involving experiential, behavioural, and physiological elements, by which the individual attempts to deal with a personally significant matter or event) | **Automatic Motivation** |
| *"I feel proud when someone says, 'I took the tablet because you explained it to me properly.'"(N1)* |  | Professional pride (Facilitator) |  |  |
| *"This strategy will work if people hear it in SHGs, schools, and from neighbors."(A1)* |  | Strategy ownership (Facilitator) | **10. Intentions**  *Conscious decision to perform behaviour* | **Reflective Motivation** |
| *"Let’s engage ward members too. If they talk in ward meetings, people will feel it’s important."(S1)* |  | Political engagement (Facilitator) |  |  |

*LF – Lymphatic Filariasis; MDA – Mass Drug Administration; COM-B – Capability, Opportunity, Motivation–Behaviour; TDF – Theoretical Domains Framework; SD – Standard Deviation. Note: Abbreviations in parentheses indicate the source of each verbatim quote – A for ASHA, N for ANM, W for Anganwadi Worker, V for Community Volunteer, T for Teacher, S for MDA Supervisor, O for NVBDCP Official, and C for Ward councillor, R for MDA Recipient*
